# Pretreatment‐Driven Modulation of Green Mango Drying: Kinetics, Energy Consumption, Techno‐Functionality, and Environmental Sustainability

**DOI:** 10.1002/fsn3.72231

**Published:** 2026-08-16

**Authors:** S. K. Fahim Tahmid Boni, S. M. Johir Rayhan, Pabitra Chandra Das, Mohammad Nurur Rahman, Farzana Haque Reeti, Md Zahir Mahmud, Md. Sajjad Hossain

**Affiliations:** ^1^ Department of Chemical Engineering Rajshahi University of Engineering and Technology Rajshahi Bangladesh

**Keywords:** drying kinetics, energy, green mango slices, hot air drying, life cycle assessment, techno‐functionality

## Abstract

Mango is a seasonal nutritious fruit, widely recognized for its rich nutritional and phytochemical profile. A huge amount of green mango spoils every year because of unfavorable environmental factors and lack of processing strategies. This study aimed to analyze the impact of different pretreatments and temperatures on drying behavior, product quality, and environmental performance of oven‐dried green mango slices. Four sample groups including control, 1% citric acid (CA), hot water blanched (HWB), and steam blanched (SB) were dried at 60°C, 65°C, and 70°C. Increasing temperature accelerated moisture removal rate in all treatments, while blanching markedly shortened the drying time, with HWB showing the highest drying rates and the shortest overall duration. Several thin‐layer models were fitted to describe the drying kinetics, and the Midilli model provided the best fit for the control sample, whereas the Modified Midilli I model best described all pretreated samples (*R* ^2^ > 0.99). Pretreatments also influenced the energy performance and functional properties of the dried samples, with blanching being the most effective one. Sensory evaluation showed improved color and flavor across all pretreated samples compared with the control. A gate‐to‐gate life cycle assessment was carried out to determine the environmental footprint and identified HWB as the lowest‐impact pretreatment within the evaluated system boundary. Overall, this study provides valuable insights on valorization of green mango for development of powder‐based functional ingredients.

## Graphical abstract

Hot‐air drying of green mango slices was evaluated following three pretreatments: citric acid (CA), hot water blanching (HWB), and steam blanching (SB). The effects of pretreatment and drying temperature were assessed through drying kinetics, thin‐layer modeling, thermodynamic properties, energy performance, product quality, and environmental impacts. HWB accelerated moisture removal, reduced drying time and specific energy consumption, and exhibited the lowest environmental impacts within the evaluated gate‐to‐gate system boundary, whereas CA provided the best sensory quality in terms of color and flavor.

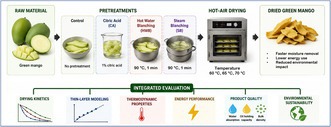

## 1 Introduction

Mango ( *Mangifera indica* L.) is a widely consumed tropical fruit known for its rich nutritional and phytochemical profile. Bangladesh cultivates ca. 205,034 ha of mango, producing 2.5 million metric tons in 2024–2025, positioning as the seventh‐largest mango producing nation worldwide (Halder and Chakma 2025). Mango usually comprises 9.8%–14.3% peel, 66.1%–72.4% pulp, 7.5%–9.3% seed coat, and 8.4%–12.4% kernels (Das et al. 2018); however, these percentages can vary depending on the climate, mango variability, season of harvest, maturity stage, and other environmental factors.

Although ripe mango is highly valued as a delicious fruit, green mango receives far less attention despite its high acidity, distinctive flavor, and potential use in intermediate‐moisture products, condiments, and functional powders. Mango is one of the most perishable produce and a large quantity of green mango gets spoiled every year due to inadequate handling, ripening, and storage conditions. Drying has emerged as one of the most effective preservation methods, as reducing water activity below 0.6 inhibits microbial growth and minimizes mechanical and physiological deterioration (Wu et al. 2024). There are several drying methods commonly used for fruits and vegetables, including freeze drying, microwave drying, infrared drying, and hot air drying. Among these, hot air drying is the most widely used technique because of its simplicity, cost‐effectiveness, and scalability; however, prolonged exposure to heat can cause enzymatic and nonenzymatic browning, nutrient degradation, and color loss (Aktas et al. 2013). Therefore, it is important to optimize the drying process to preserve the quality of fruits and vegetables.

Previous studies have shown that pretreatments like blanching, soaking in osmotic solution, ultrasound, pulsed electric field, cold plasma, and high‐pressure treatments can modify the tissue structure and improve mass transfer, which can have an impact on the drying efficiency and the quality of the final dried product (Bi et al. 2015). Among the different pretreatment techniques, blanching has been widely recognized as an efficient approach to reduce drying time and inactivate enzymes, but excessive blanching may lead to significant cellular damage and phytochemical loss (Wang et al. 2018, 2021). Beyond heat‐based treatments, chemical inhibitors such as sulfites have traditionally been used to control enzymatic browning but growing health concerns and regulatory restrictions on their use in fresh produce have increased the need for safer alternatives (Sivakumar et al. 2010). Citric acid has become a safer alternative as it can prevent enzymatic browning, suppress microbial growth, and prolong the shelf life of fresh produce (Bata Gouda et al. 2021). Therefore, citric acid continues to be a preferred pretreatment in drying studies, thanks to its multifunctional properties and regulatory acceptance.

Designing, optimizing, and managing the drying process requires a thorough understanding of the drying properties and heat and mass transport phenomenon inside the agricultural product. Mathematical thin layer models of the drying process also play an important role to predict drying performance as these models help to describe the moisture removal pattern and provide a reliable basis to evaluate the process parameters, improve dryer design, and maintain product quality. Because of its extensive versatility, thin‐layer models are now widely used in both research and industrial applications to analyze and optimize drying operations. Alongside process efficiency, dried fruit powders must meet functional and sensory expectations for successful industrial application. Pretreatments can significantly influence techno‐functional properties by altering cell structure, pigment stability, and phytochemical retention.

Furthermore, environmental sustainability has also become central to modern food processing research and life cycle assessment (LCA) has become a standard approach for measuring environmental performance and identifying hotspots throughout a product's life cycle. Recent studies have successfully applied LCA to assess sustainability in various sectors, including sewage sludge drying (Karadirek et al. 2025), nature‐based coagulant (Cojbasic et al. 2025), crude peptides from green coffee beans (Hunsub et al. 2024), woody biomass (Edmonson et al. 2025), and milk powder production (Zhang et al. 2021). These examples demonstrate the versatility of LCA and its importance as a decision‐support tool to develop cleaner and more efficient processing methods. Therefore, integrating LCA into drying research can provide an in‐depth understanding of process optimization.

Although several studies have investigated the drying kinetics and quality characteristics of mango products, most have focused on ripe mango, individual pretreatment techniques, or conventional quality parameters with limited attention to process efficiency and environmental sustainability. However, studies integrating drying kinetics, thermodynamic behavior, energy performance, techno‐functional properties, sensory quality, and environmental sustainability assessment for green mango drying are still lacking. In addition, comparative investigations of chemical pretreatment (citric acid) and physical pretreatments (hot water and steam blanching) under identical hot‐air drying conditions have rarely been conducted for green mango slices. Therefore, this study evaluates drying behavior, thin‐layer modeling, energy performance, techno‐functional properties, and gate‐to‐gate environmental impacts to identify suitable pretreatment strategies for green mango valorization. The main goal of this work is to provide comprehensive insights to guide the sustainable and value‐driven utilization of green mango.

## 2 Materials and Methods

### 2.1 Raw Materials and Sample Preparation

Fresh matured mangoes (BARI Aam‐9) were collected from a local mango garden in Charghat, Rajshahi, Bangladesh (24.2833° N 88.7750° E). Fruits were selected based on uniform mature‐green appearance, firmness, and absence of visible defects. All samples were sourced from a single harvesting batch on the same day to ensure uniformity in maturity, cultivar characteristics, and pre‐harvest environmental conditions. This strategy was adopted to reduce biological variability and enable a controlled comparison of pretreatment and drying temperature effects. After collection, the mangoes were cleaned properly before peeling and then sliced into a uniform thickness of 2.5 mm. The sliced samples were then randomly assigned to the different pretreatment groups as described in Section 2.2 and immediately subjected to their respective pretreatments before drying to minimize enzymatic deterioration.

### 2.2 Pretreatments

Four sample groups were prepared – (a) control (untreated), (b) soaking in a citric acid (1% w/v) solution (CA), (c) hot water blanching (HWB), and (d) steam blanching (SB). One percent CA solution was chosen based on previous fruit drying studies showing that concentrations within the 0.5%–2% range effectively reduce enzymatic browning and preserve color, with 1% commonly used as a practical and nondestructive level (Nguyen et al. 2025; Nyangena, Owino, Ambuko, and Imathiu 2019; Nyangena, Owino, Imathiu, and Ambuko 2019). pH after CA treatment was not directly measured in this study. For CA samples, sliced samples were dipped into the acidic solution for 30 min and then blotted with a clean dry sheet to remove the external moisture. SB was performed using a thermostatic water bath (Model HJ‐A4, Hinotek, China), while HWB was carried out using a boiling pan and gas stove at 90°C ± 2°C; both were blanched for 1 min. The blanching condition was selected based on preliminary observations and previously reported fruit drying studies, where short‐duration blanching was sufficient to improve drying performance and enzyme inactivation while minimizing excessive thermal softening and phytochemical degradation (Xiao et al. 2017). After blanching, the blanched samples were dipped into cold water (4°C) immediately for lowering the temperature. Prior to drying, the initial moisture content of the mango slices was determined using the AOAC oven‐drying method (Horwitz 2005) and was 76.15% ± 1.24% (wet basis), corresponding to 3.19 ± 0.23 g water g^−1^ dry matter.

### 2.3 Drying Experiments

Drying was performed at 60°C, 65°C, and 70°C using a convective oven dryer (Model DHG‐9075A). These temperature levels were selected based on commonly reported ranges for convective drying of fruit materials in previous studies (Iqbal et al. 2019; Kumar et al. 2014; Mishra et al. 2021). The air velocity was automatically controlled by the instrument at a factory‐set air velocity of approximately 1 m s^−1^, which could not be externally adjusted during the experiments. Samples were uniformly spread on the tray in a single layer without overlapping to ensure thin‐layer drying conditions and uniform exposure to circulating hot air.

Once the dryer reached the target temperature, the samples were placed on separate trays and kept within the cabinet. For each treatment–temperature combination, a single batch of mango slices was dried and monitored continuously, and the sample mass was recorded at 30 min intervals during the initial drying stage (first hour) and subsequently at 60 min intervals until equilibrium moisture content was achieved. Mass loss was determined gravimetrically from the difference between the initial sample mass and the mass recorded at predetermined drying intervals, and the data were used to determine the drying rate and moisture ratio. The dried samples were then milled using a grinder to convert into powder and passed through a 30‐size standard mesh sieve to obtain the fine powder.

### 2.4 Evaluation of Drying Characteristics

#### 2.4.1 Moisture Ratio and Drying Rate

Once the sample reaches equilibrium moisture level, the drying experiments were stopped. Moisture loss was recorded at regular intervals. The moisture ratio (MR) was calculated according to Equation (1) as mentioned by Gebeyehu et al. (2025):

(1) $$\mathrm{MR}=\frac{M_{t}-M_{e}}{M_{0}-M_{e}}$$

where $M_{t}$ is the moisture content (db) at time t (h), $M_{0}$ is the initial moisture content (db) of the sample, and $M_{e}$ is the equilibrium moisture content (db). Since the values of $M_{0}$ and $M_{t}$ were considerably higher than $M_{e}$ throughout the drying process, Equation (1) was simplified to Equation (2) (Wang et al. 2021),

(2) $$\mathrm{MR}=\frac{M_{t}}{M_{0}}$$

The DR of the green mango samples was expressed in g water g^−1^ dry matter per min and calculated using the relation described by Equation (3) (Kumar et al. 2019):

(3) $$\mathrm{DR}=\frac{M_{t}-M_{t+\mathrm{dt}}}{\mathrm{dt}}$$

where $M_{t+\mathrm{dt}}$ is the moisture content at time *t + dt*, and dt is the time interval between two consecutive measurements.

#### 2.4.2 Thin Layer Mathematical Modeling

In this experiment, 18 different mathematical models were used to characterize the drying behavior of the four sample groups as presented in Table 1. The models were further fitted to the experimental MR data using nonlinear regression and the model suitability was evaluated based on coefficient of determination ($R^{2}$), chi‐squared statistic ($\chi^{2}$), sum of squared errors (SSE), and root mean square error (RMSE). The maximum *R* ^2^ values and the minimum *χ* ^2^ and RMSE, suggest the optimal goodness of fit (Chigbo et al. 2024). The equations for calculating *R* ^2^, *χ* ^2^, SSE, and RMSE are shown in Equations ((4), (5), (6), (7))–((4), (5), (6), (7)).

(4) $$R^{2}=\frac{\sum_{i=1}^{N}{\mathrm{MR}}_{\mathrm{Pre},i}{\mathrm{MR}}_{\mathrm{Exp},i}-\sum_{i=1}^{N}{\mathrm{MR}}_{\mathrm{Pre},i}\sum_{i=1}^{N}{\mathrm{MR}}_{\mathrm{Exp},i}}{\sqrt{\left(\sum_{i=1}^{N}{\left({\mathrm{MR}}_{\mathrm{Pre},i}\right)}^{2}-\left(\sum_{i=1}^{N}{\left({\mathrm{MR}}_{\mathrm{Pre},i}\right)}^{2}\right)\;(N\sum_{i=1}^{N}{\mathrm{MR}}_{\mathrm{Exp},i}-{\left(\sum_{i=1}^{N}{\mathrm{MR}}_{\mathrm{Exp},i}\right)}^{2}\right)}}$$

(5) $$\chi^{2}=\frac{\sum_{i=1}^{n}{\left({\mathrm{MR}}_{\mathrm{Exp},i}-{\mathrm{MR}}_{\mathrm{Pre},i}\right)}^{2}}{N-z}$$

(6) $$\mathrm{SSE}=\sum_{i=1}^{N}{\left(y_{i}-{\hat{y}}_{i}\right)}^{2}$$

(7) $$\text{RMSE}=\sqrt{\left[\sum_{i=1}^{N}\frac{1}{N}\;{\left({\mathrm{MR}}_{\mathrm{Exp},i}-{\mathrm{MR}}_{\mathrm{Pre},i}\right)}^{2}\right]}$$

where MR_Exp,*i*_ is the MR (experimental), MR_Pre,*i*_ is the MR (predicted), *N* is the observation number, *z* is the number or parameters, $y_{i}$ represents the experimental value, and ${\hat{y}}_{i}$ denotes the predicted value from the model.

**TABLE 1:** Different thin‐layer mathematical models and their equations used in this study.

No.	Model name	Model equation	Model constants	References
1	Newton	MR = exp (−*kt*)	k	Chigbo et al. (2024)
2	Page	MR = exp (−*kt* ^*n*^)	k, n	Chigbo et al. (2024)
3	Modified Page	MR = exp [−(*kt*)^*n*^]	k, n	Chigbo et al. (2024)
4	Henderson and Pabis	MR = *a* exp (−*kt*)	k, a	Gebeyehu et al. (2025)
5	Midilli	MR = *a* exp(−*kt* ^*n*^) *+ bt*	k, a, n, b	Buzrul (2022)
6	Modified Midilli I	MR = *a* exp (−*kt* ^*n*^) *+ b*	k, n, a, b	Buzrul (2022)
7	Modified Midilli II	MR = exp(−*kt* ^*n*^) *+ bt*	k, n, b	Buzrul (2022)
8	Logarithmic	MR = *a* exp (−*kt*) *+ c*	k, a, c	Gebeyehu et al. (2025)
9	Wang‐Singh	MR = 1 + *at + bt* ^*2*^	a, b	Buzrul (2022)
10	Weibullian	MR = exp (−${\left(\frac{t}{\alpha }\right)}^{\beta }$)	*α,β*	Elwakeel et al. (2025)
11	Weibullian I	MR = $10^{-{\left(\frac{t}{\delta }\right)}^{n}}$	δ, n	Elwakeel et al. (2025)
12	Peleg	MR = 1 − $\frac{t}{\left(a+\mathrm{bt}\right)}$	a, b	Inyang et al. (2018)
13	Silva and Others	MR = exp (−*at − b* $\sqrt{t}$)	a, b	da Silva et al. (2014)
14	Cubic	MR = *at* ^3^ *+ bt* ^2^ *+ ct + d*	a, b, c, d	Çerçi and Sufer (2018)
15	Demir et al.	MR = *a* exp (*−kt*) ^*n*^ *+ b*	k, a, n, b	Chigbo et al. (2024)
16	Binomial	MR = *at* ^*2*^ *+ bt + c*	a, b, c	Inyang et al. (2018)
17	Two‐term	MR = *a* exp (−*kt*) + *b* exp (−*k*_1_ *t*)	k, k_1_, a, b	Buzrul (2022)
18	Hii and Others	MR = *a* exp (−*kt* ^*n*^) + *b*exp (*−k*_1_ *t* ^*n*^)	k, k_1_, a, b, n	Chigbo et al. (2024)

#### 2.4.3 Effective Moisture Diffusivity and Activation Energy

The changes in the moisture concentration were evaluated to determine the effective moisture diffusivity (*D*_eff_), expressed in m^2^s^−1^, using Equation (8), derived from Fick's second law of diffusion (I. Doymaz 2008).

(8) $$\mathrm{MR}=\frac{8}{\pi^{2}}\sum_{n=0}^{\propto }\frac{1}{{\left(2n+1\right)}^{2}}\exp\left(\frac{-{\left(2n+1\right)}^{2}\pi^{2}D_{\mathrm{eff}}t}{4L^{2}}\right)$$

The linearized form of the equation was obtained by plotting ln (MR) against drying time (h) as shown in Equation (9).

(9) $$\ln\mathrm{MR}=\ln\frac{8}{\pi^{2}}-\frac{\pi^{2}D_{\mathrm{eff}}}{4L^{2}}t$$

where *L* is the half‐thickness of the mango slice (m). The slope (m) of this straight line was used to calculate *D*_eff_ as described by Yamchi et al. (2024).

(10) $$m=-\frac{\pi^{2}D_{\mathrm{eff}}}{4L^{2}}$$

Activation energy (*E*_a_) was determined using the Arrhenius equation, as described by Tunckal and Doymaz (2020). To establish a linear relationship, the natural logarithm was applied to both sides of the Arrhenius equation. By plotting ln(*D*_eff_) against the reciprocal of the absolute temperature (1/T), a straight line was obtained, whose slope ($K_{0}$) enables the calculation of *E*_a_ (Okunola et al. 2023).

(11) $$D_{\mathrm{eff}}=D_{0}\;\exp^{-\frac{E_{a}}{\mathrm{RT}}}$$

(12) $$\ln D_{\mathrm{eff}}=\ln D_{0}-\frac{E_{a}}{R}\left(\frac{1}{T}\right)$$

(13) $$K_{0}=-\frac{E_{a}}{R}$$

where T is the absolute temperature (K), $D_{0}$ is the frequency factor (m^2^s^−1^), and R is the universal gas constant (8.3143 J mol^−1^ K^−1^).

#### 2.4.4 Thermodynamic Parameters

The *E*_a_ was used to determine various thermodynamic parameters, including the enthalpy (Δ*H*), entropy (ΔS), and Gibbs free energy (Δ*G*), calculated using Equations ((14), (15), (16))–((14), (15), (16)), respectively, according to the approach reported by de Jesus Junqueira et al. (2024).

(14) $$\Delta H=E_{a}-\mathrm{RT}$$

(15) $$\Delta S=R\;(ln\;D_{0}-ln\;(\frac{k_{b}}{h})-ln\;T)$$

(16) ΔG=ΔH−TΔS

where *E*_a_ is the activation energy (kJmol^−1^), h = 6.6262 × 10^−34^ J.s is the Planck's constant, and $k_{b}$= $1.3806\times 10^{-23}\;J\;K^{-1}$ is the Boltzmann's constant. ΔH and ΔG are reported in kJ mol⁻¹, while ΔS is reported in J mol⁻¹ K⁻¹.

### 2.5 Energy Analysis

The impacts of different drying parameters on energy efficiency were defined as the ratio between the heat energy used and the total heat energy supplied by the oven was determined using Equation (17), following the approach of Chahbani et al. (2023). Moreover, the specific energy consumption (SEC), expressed in kWhkg^−1^ of water, represents the energy required to evaporate 1 kg of water under a given drying temperature during the drying period, using an average dryer power of 0.5 kW. It was determined using Equation (18) (Rasooli Sharabiani et al. 2021).

(17) $$\eta \left(\%\right)=\frac{m_{w}h_{\mathrm{fg}}}{\mathrm{Pdt}}\times 100$$

(18) $$\mathrm{SEC}\;\left(kWh\;{\mathrm{kg}}^{-1}\;\text{water}\right)=\frac{\mathrm{Pt}}{m_{w}}$$

where *m*_w_ is the mass of water evaporated (kg), *h*_fg_ is the latent heat of vaporization (J kg^−1^), *P* is the average dryer power (kW), and t is the drying time (s).

In addition to SEC, moisture extraction rate (MER) and specific moisture extraction rate (SMER) were calculated. MER describes the rate at which water is extracted from the material over time and SMER represents the amount of moisture removed per unit of energy consumed during drying, calculated using Equations (19) and (20), respectively, as provided by Baysal et al. (2015),

(19) $$\mathrm{MER}=\frac{\Delta m_{w}}{\Delta t}$$

(20) $$\text{SMER}=\frac{\mathrm{\Delta DR}}{P\Delta t}$$

where ΔDR is the amount of moisture removed (kg of water) and Δt represents the drying time (h).

### 2.6 Techno‐Functional Properties and Total Phenolic Content

#### 2.6.1 Bulk Density

A 15 mL cylinder was carefully filled with green mango powder and carefully weighed. The bottom was tapped lightly on the laboratory bench multiple times after filling the cylinder until the sample had completely stopped decreasing. The mass (g) of the sample was divided by the volume (cm^3^) to calculate the bulk density using Equation (21) (Das et al. 2019).

(21) $$\text{Bulk Density}\;\left(g\;{\mathrm{cm}}^{-3}\right)=\frac{\text{weight}\;\left(g\right)\;}{\text{volume}\;\left({\mathrm{cm}}^{3}\right)}$$

#### 2.6.2 Water Absorption Capacity

About 0.5 g of green mango powder was weighed, and 5 mL of distilled water was added to a centrifuge tube. The mixture was agitated for 30 s to ensure uniform wetting. Then, the tube was centrifuged at 3000 rpm for 30 min (Chandra et al. 2015; Vaishali et al. 2020). Following the centrifugation, the supernatant water was carefully drained, and each tube was reweighed. The water absorption capacity (WAC), expressed as g of water absorbed per g of dried powder, was calculated as follows (Badiora et al. 2023).

(22) $$\begin{array}{c} \mathrm{WAC}\;\left(g\;\text{water}\;g^{-1}\;\text{powder}\right)=\\ \frac{\left(\mathrm{wt}\;\text{of tube after draining of water}-\mathrm{wt}\;\text{of}\;\mathrm{dry}\;\text{tube}\right)-\mathrm{wt}\;\text{of total powder}}{\mathrm{wt}\;\text{of total powder}} \end{array}$$

#### 2.6.3 Oil Absorption Capacity

The oil absorption capacity (OAC) was determined by following a previously reported method (Das et al. 2019), with slight modification. About 0.5 g of dried mango powder was weighed and added with 5 mL of vegetable oil into a pre‐weighed centrifuge tube. The mixture was then stirred for 1 min for complete dispersion. After this, the tube was left at ambient temperature for 30 min and then centrifuged at 3000 rpm for 30 min (Chandra et al. 2015; Vaishali et al. 2020). After centrifugation, the separated oil was carefully removed, and tube was inverted onto oil‐absorbent paper for 25 min to allow any remaining surface oil to drain before reweighing. The OAC was expressed as g of oil absorbed per g of dried powder and calculated using Equation (23).

(23) $$\begin{array}{c} \mathrm{OAC}\;\left(g\;\text{oil}\;g^{-1}\;\text{powder}\right)=\\ \frac{\left(\mathrm{wt}\;\text{of tube after draining of oil}-\mathrm{wt}\;\text{of}\;\mathrm{dry}\;\text{tube}\right)-\mathrm{wt}\;\text{of total powder}}{\mathrm{wt}\;\text{of total powder}} \end{array}$$

#### 2.6.4 Total Phenolic Content

The extraction of the phenolic compounds from dried mango powder was carried out by following the procedure described by (León‐Roque et al. 2023). Briefly, 0.5 g of dried mango powder was extracted with 10 mL of methanol/water (80:20, v/v) under continuous stirring for 30 min at room temperature. The mixture was centrifuged at 5000 × *g* for 15 min at 4°C, and the supernatant was collected for analysis. TPC was quantified using the Folin–Ciocalteu (FC) method adapted from Fernández‐Estellé et al. (2023). The absorbance was measured at 765 nm using a UV–Vis spectrophotometer (Shimadzu UV‐3600i Plus, Japan), and the results were expressed as mg gallic acid equivalents (GAE) per 100 g of dried mango powder (mg GAE/100 g DW).

### 2.7 Sensory Evaluation

The sensory evaluation of the dried mango powder was conducted with a semi‐trained panel of 10 participants who provided informed consent. Before the evaluation, panelists were briefed on sensory terminology and familiarized with the evaluation procedures based on the guidelines outlined by Jowitt (2007) and the samples were presented using random three‐digit codes in randomized order to ensure blind evaluation. The evaluation focused on color and flavor, as these are among the most important attributes affecting consumer perception of dried fruit products. Each panelist evaluated powders obtained from four different pretreatments and for each pretreatment, samples produced at three drying temperatures were evaluated. The average scores of the panelists were calculated to represent the final sensory scores for each treatment. A nine‐point hedonic scale was used to evaluate color and flavor, where 9 = like extremely, 8 = like very much, 7 = like moderately, 6 = like slightly, 5 = neither like nor dislike, 4 = dislike slightly, 3 = dislike moderately, 2 = dislike very much, and 1 = dislike extremely (Chong et al. 2025).

### 2.8 Life Cycle Assessment

The first step toward making food production more sustainable is to understand the environmental footprint of the product. This is usually carried out through LCA, which helps to compare different production methods and determine the hotspots that contribute most to environmental impact. To conduct a comprehensive LCA study, it is important to define the scope by selecting a functional unit and establishing the system boundaries. Another crucial step is to gather the necessary inventory and process data required for life‐cycle inventory (LCI) development. And finally, an appropriate LCA method is utilized to estimate emissions to water, air, and soil.

#### 2.8.1 Goal and Scope

**FIGURE 1:**
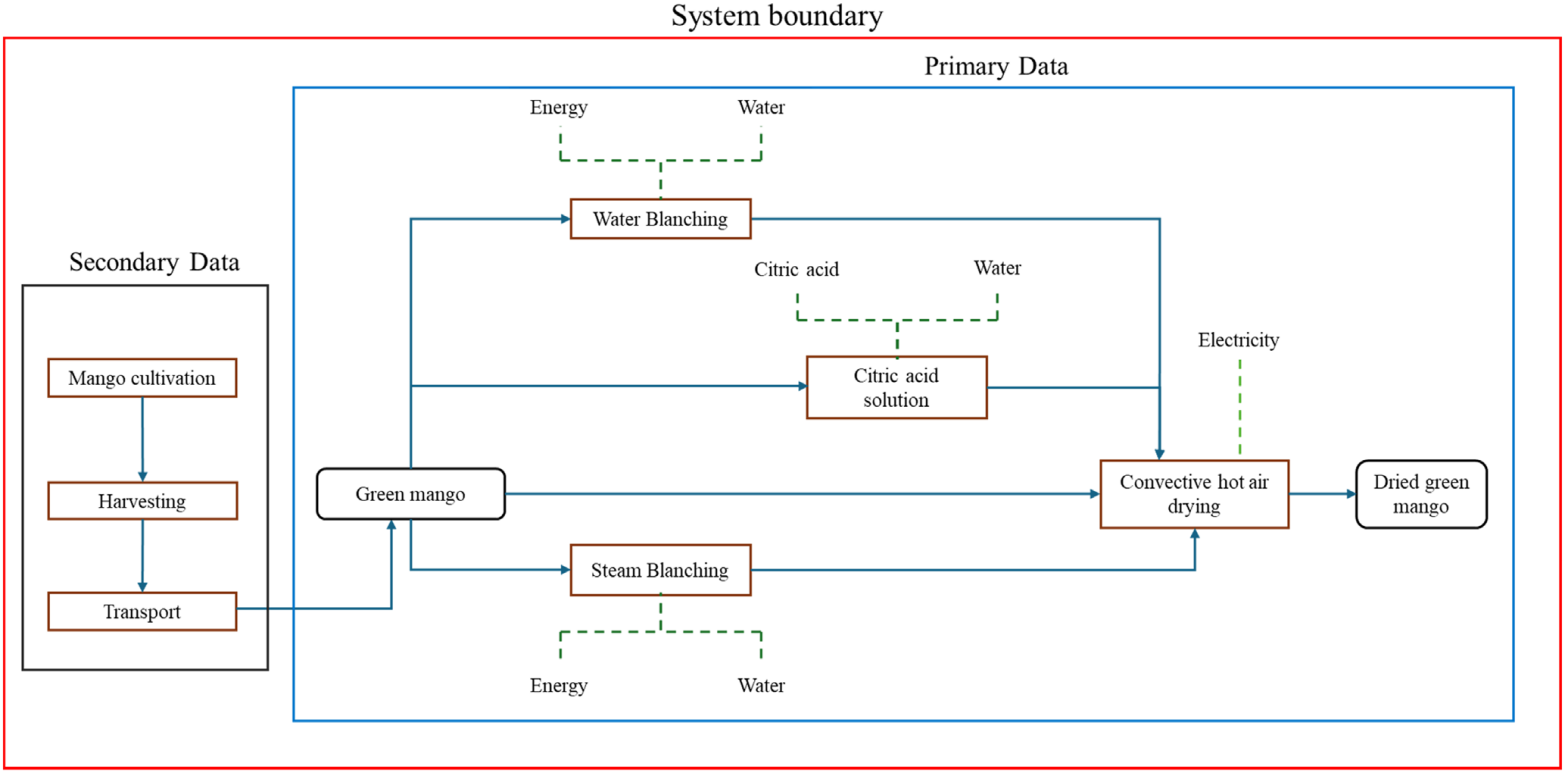
System boundary for the production of dried green mango under different pretreatment conditions using a gate‐to‐gate life cycle assessment approach. The black‐bordered boxes represent upstream processes (mango cultivation, harvesting, and transportation) modeled using secondary inventory data, whereas the blue‐bordered process pathways represent primary experimental data collected during laboratory‐scale pretreatment and drying operations. Green dashed lines indicate auxiliary energy and material inputs associated with each processing stage.

Defining the goal is the first step in any LCA, as it establishes the scope of the assessment and the reference quantity used throughout the study. The LCA methodology applied in this study follows the framework described in ISO 14040 and ISO 14044 standards, which provide guidelines for goal and scope definition, life cycle inventory analysis, impact assessment, and interpretation (ISO 2006a, 2006b; Hauschild et al. 2018). The assessment focused exclusively on the drying stage because it represents the most energy‐intensive and variable part of the processing chain. In this study, a gate‐to‐gate boundary was applied, and the functional unit was set as 1 kg of dried green mango. Background processes (such as agricultural production) were included using secondary data to ensure complete and consistent life‐cycle inventories. As all samples were sourced from the same batch of fresh produce, the infrastructure and transportation requirements were considered equivalent for all the samples. The system boundary is shown in Figure 1.

#### 2.8.2 Life Cycle Inventory

The materials and components of the drying system, along with their masses and volumes, are given in Table S1. Primary data for the drying stages were obtained directly from laboratory experiments. Secondary data of the mango cultivation were gathered from several sources (Basset‐Mens et al. 2018; Ram and Verma 2015; World Population Review 2025), then converted to match the functional unit of this study. Background inventory data for electricity, water, chemicals, and other upstream inputs were taken from the Ecoinvent v3.9.1 database.

#### 2.8.3 Life Cycle Impact Assessment

The ReCiPe 2016 (H) method implemented in SimaPro 9.0.0.49 was selected for environmental impact assessment. The hierarchist perspective was chosen because it reflects commonly accepted scientific assumptions regarding time horizon and environmental impact mechanisms and is widely adopted in studies using the ReCiPe2016 life cycle impact assessment framework (Huijbregts et al. 2017). Eighteen midpoint indicators were evaluated to provide a detailed understanding of potential environmental burdens throughout the cycle.

### 2.9 Statistical Analysis

Drying curves and data analyses were generated using OriginPro 2025b (Learning Edition). Nonlinear regression and data handling were performed using the Solver add‐in in Microsoft Excel 2016. Drying experiments were conducted as single continuous thin‐layer runs for each treatment‐temperature combination, with measurements collected throughout drying for kinetic modeling. Statistical analyses were applied only to replicated TPC, techno‐functional property, and organoleptic characteristics, which were analyzed in triplicate using one‐way ANOVA followed by Tukey's HSD test in IBM SPSS, with results expressed as mean ± standard deviation.

## 3 Results and Discussion

### 3.1 Effect of Temperature and Pretreatment on Drying Characteristics

**FIGURE 2:**
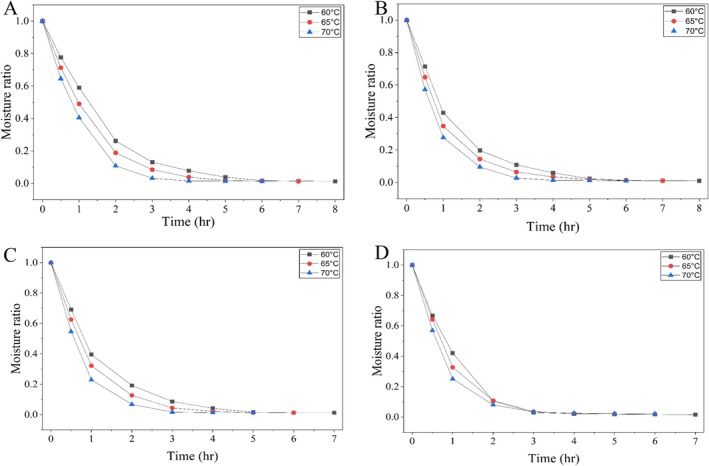
Drying curves of green mango slices at different drying temperature levels showing dimensionless moisture ratio (MR) against drying time for control (A), 1% citric acid treated (B), hot water blanched (C), and steam blanched (D) samples.

Figure 2A–D shows the effect of drying temperature on the MR over time. Across all treatments, the increasing drying temperature accelerated the moisture removal, suggesting the strong influence of temperature on the drying behavior of green mango slices. MR declined rapidly in the early stages of drying, followed by a gradual decline as the process progressed.

**FIGURE 3:**
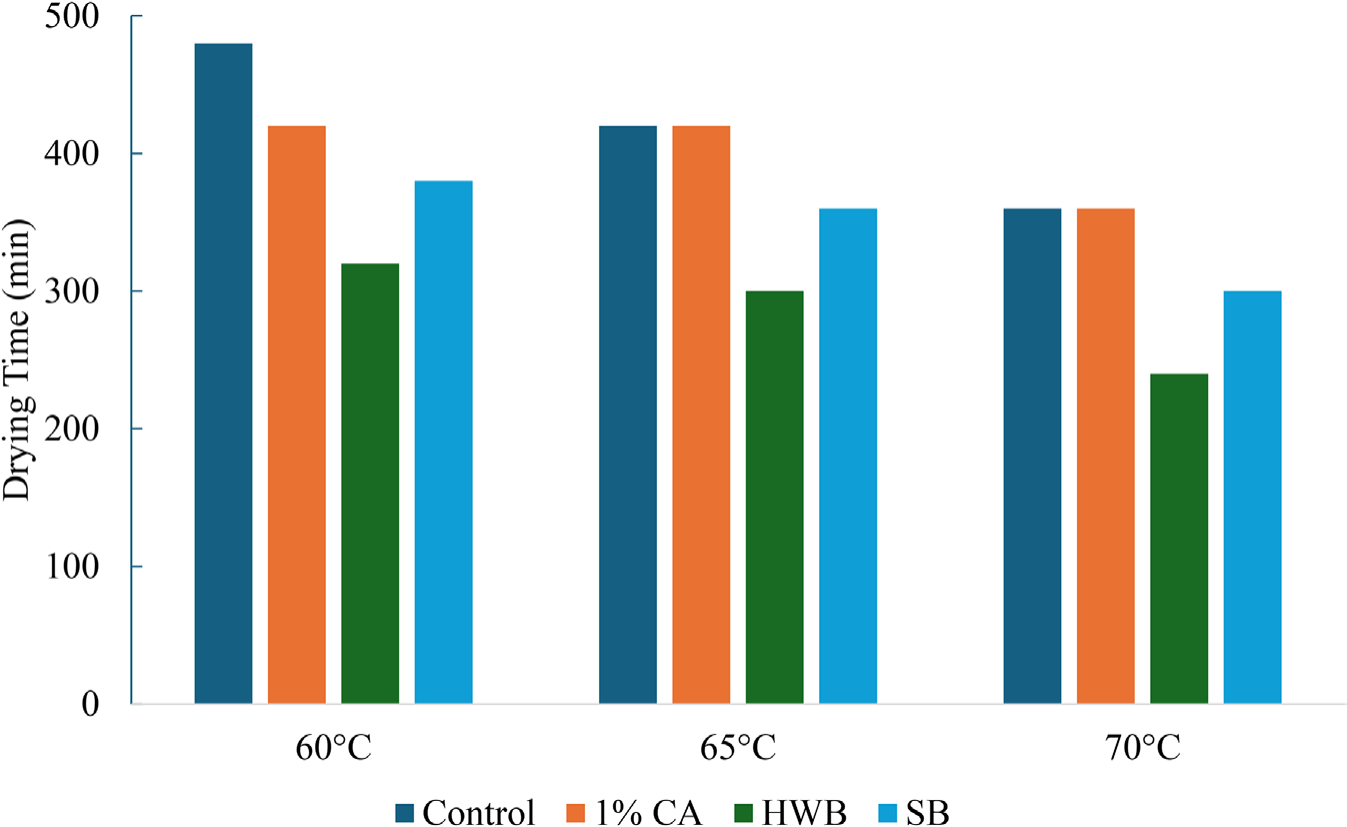
Drying time required by each pretreated sample to reach the equilibrium level. CA, citric acid; HWB, hot water blanching; SB, steam blanching.

In terms of pretreatment, blanching effectively reduced the drying time compared to both CA and control samples (Figure 3). Relative to the control, HWB reduced the total drying time by approximately 3 h at 60°C and 2 h at both 65°C and 70°C. Similarly, SB reduced the drying time by approximately 2 h at 60°C and 1 h at both 65°C and 70°C. This behavior may be linked to thermal disruption of cell wall polysaccharides and increased porosity, which lowers internal mass transfer resistance and opens pathways for moisture to move more easily from the interior to the surface, thereby shortening the overall drying time (Yuan et al. 2023). Similar results have also been reported in previous drying studies where blanching pretreatment enhanced moisture diffusion and reduced drying time in agricultural products (Adetoro et al. 2020; An et al. 2022).

**FIGURE 4:**
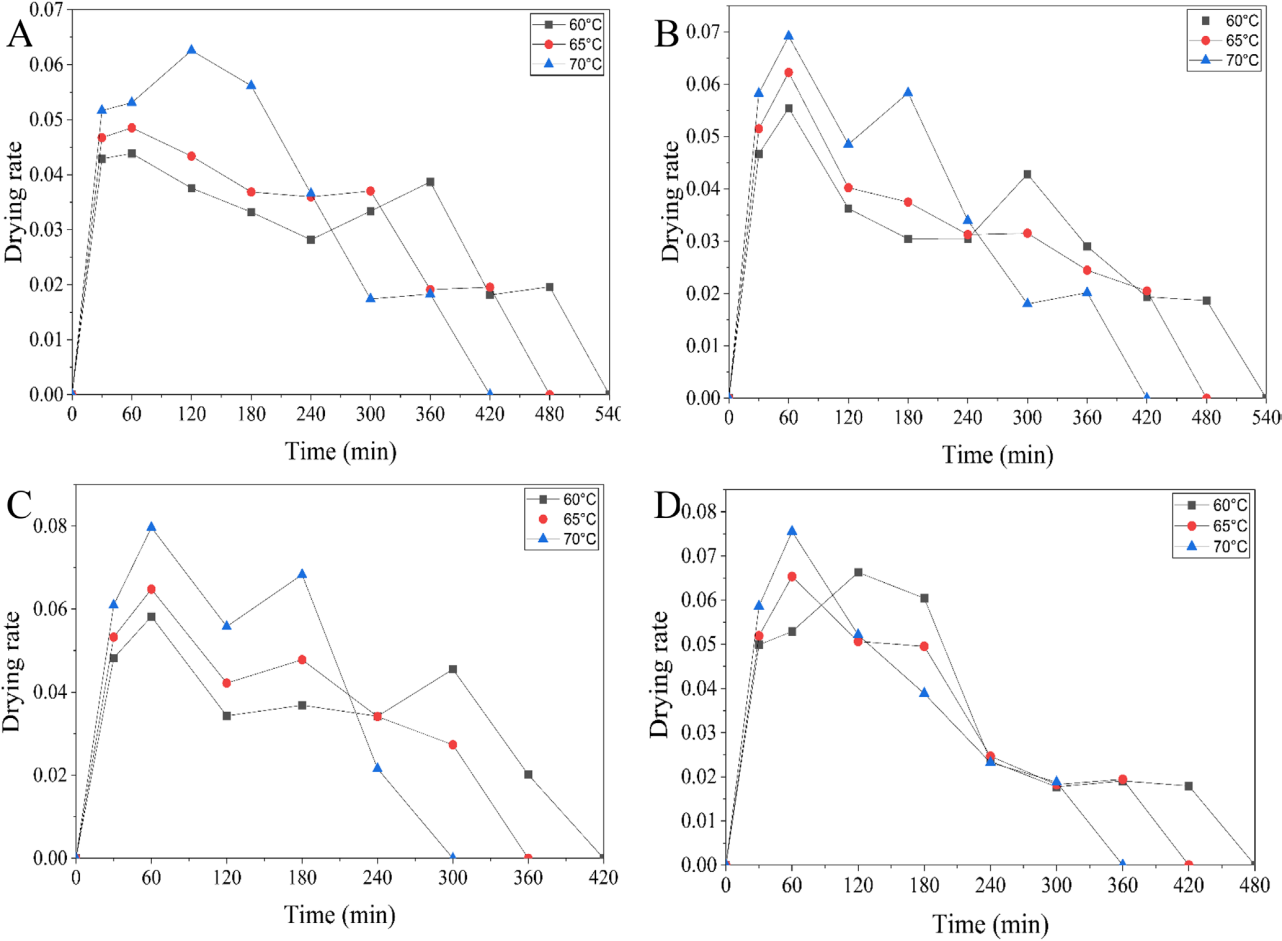
Effect of temperature and pretreatments on drying rate of green mango slices for control (A), 1% citric acid treated (B), hot water blanched (C), and steam blanched (D) samples.

The drying rate curves in Figure 4A–D show the influence of temperature and pretreatments on moisture removal kinetics during hot air drying. In all treatments, the drying rate increased sharply at the beginning of the process and then declined steadily as internal diffusion became the dominant mechanism. This behavior is commonly exhibited by biological materials, where internal resistance to moisture transfer increases as drying progresses (Tunde‐Akintunde and Ogunlakin 2013). At the early stage of drying, free water located near the surface evaporates rapidly, leading to a high drying rate. As drying proceeds, the removal of bound water from the internal structure becomes more difficult, resulting in a progressive decline in drying rate (Zeng et al. 2024).

Higher temperatures consistently produced higher drying rates, which may be attributed to stronger vapor pressure gradients that ultimately accelerate moisture diffusion and reduce viscosity within cell matrices, resulting in rapid migration of bound water (Erbay and Icier 2010). For instance, the 70°C curves for all treatments reached their maximum rate earlier and declined more quickly, indicating that higher drying temperatures are more efficient for internal water transportation. This trend aligns with previous findings in hog plum (Ojediran et al. 2021) and kiwifruit (İ. Doymaz 2020), where drying rate and effective moisture diffusivity increased significantly with temperature due to the exponential dependence of diffusivity on thermal energy.

Pretreatments also had a significant influence on drying behavior. Among the treated samples, HWB showed higher drying rates than other samples during the early and middle stages of drying. SB followed a similar pattern but with slightly lower rates, likely because it is a milder process and causes less extensive tissue softening. This pattern suggests that blanching enhanced moisture transport within the tissue, resulting in higher drying rates during the early and middle drying stages. Falade and Shogaolu (2010) observed that blanching significantly enhanced the drying rate of pumpkin slices, possibly due to breakdown of cell wall, while Barathiraja et al. (2022) reported similar effects during the drying of turkey berry. These findings suggest that the variation in drying rate among different pretreatments and temperatures is primarily governed by differences in internal moisture diffusivity and structural alterations caused by thermal and chemical treatments.

### 3.2 Thin‐Layer Model Fitting

In the drying process, the interplay between heat transfer and mass transfer phenomena is a fundamental aspect and it is important to conduct a comprehensive evaluation based on engineering principles to fully understand the drying affecting factors (Tuly et al. 2023). Therefore, the MR of different pretreated samples at 60°C, 65°C, and 70°C was fitted to thin layer drying models to assess and compare drying behavior.

All evaluated drying models produced *R* ^2^ values greater than 0.97 across the different pretreatment conditions (Tables S2–S5), which suggests a strong correlation between experimental and predicted data (I. Doymaz 2008). Among the tested models, the Midilli‐type models consistently yielded the highest *R* ^2^ values together with the lowest RMSE and *χ* ^2^ values across all drying conditions (Table 2). Compared with Page, Henderson and Pabis, and Logarithmic models, Midilli‐type models better described both the initial rapid moisture removal and subsequent diffusion‐controlled drying stages because they incorporate both exponential and linear terms (Midilli et al. 2002). This greater modeling flexibility might contribute to their superior predictive performance for pretreated green mango slices, where pretreatment‐induced structural modifications influenced internal moisture transport behavior. Similar observations regarding the strong suitability of Midilli‐type models for agricultural materials have also been reported for mango and banana drying by Mugodo and Workneh (2021) and Tunckal and Doymaz (2020), respectively. The validation of the selected models is further demonstrated in Figures S1–S4. Therefore, the MR of the various samples at different drying stages (60°C–70°C) can be accurately estimated using the equations:

(24) $$\text{Control}:\mathrm{MR}=0.999\;\exp\left(-0.749\times t^{1.17}\right)+\text{2E}-3\times t$$

(25) $$1\%\text{citric acid}:\mathrm{MR}=0.987\;\exp\left(-1.029\times t^{1.09}\right)+1.\text{6E}-2$$

(26) $$\mathrm{Hot}\;\text{water blanched}:\mathrm{MR}=0.984\;\exp\left(-1.555\times t^{1.14}\right)+1.\text{8E}-2$$

(27) $$\text{Steam blanched}:\mathrm{MR}=0.978\;\exp\left(-1.159\times t^{1.25}\right)+2.\text{2E}-2$$

**TABLE 2:** Statistical results of best fitting models across different pretreated samples.

Sample	Best model	Temp (°C)	Parameters				SSE	*R* ^2^	RMSE	*χ* ^2^
			*k*	*a*	*b*	*n*				
Control	Midilli	60	0.5604	1.0000	0.0020	1.1927	0.0011	0.9991	0.0104	0.0002
		65	0.7363	0.9992	0.0020	1.1499	0.0003	0.9997	0.0057	0.0001
		70	0.9507	0.9974	0.0019	1.1830	0.0006	0.9994	0.0088	0.0002
CA	Modified Midilli I	60	0.8079	0.9883	0.0171	1.0676	0.0021	0.9979	0.0154	0.0004
		65	1.0160	0.9855	0.0180	1.0885	0.0016	0.9984	0.0133	0.0003
		70	1.2637	0.9882	0.0132	1.1082	0.0007	0.9993	0.0091	0.0002
HWB	Modified Midilli I	60	0.8681	0.9890	0.0155	1.0594	0.0022	0.9976	0.0167	0.0006
		65	1.1043	0.9830	0.0194	1.1258	0.0012	0.9986	0.0130	0.0004
		70	1.4940	0.9811	0.0199	1.2421	0.0006	0.9992	0.0101	0.0003
SB	Modified Midilli I	60	0.9313	0.9824	0.0142	1.2719	0.0008	0.9992	0.0097	0.0002
		65	1.1215	0.9782	0.0238	1.2411	0.0006	0.9994	0.0084	0.0001
		70	1.4257	0.9718	0.0293	1.2467	0.0006	0.9993	0.0092	0.0002

Abbreviations: $\chi^{2}$, chi‐squared statistic; CA, citric acid; HWB, hot water blanched; *R* ^2^, coefficient of determination; RSME, root mean square error; SB, steam blanched; SSE, sum of squared errors; Temp, temperature.

### 3.3 Effective Moisture Diffusivity and Thermodynamic Parameters

Table 3 presents the drying rate constant (*k*), effective moisture diffusivity (*D*_eff_), *R* ^2^, activation energy (*E*_a_), and thermodynamic properties (Δ*H*, Δ*G*, and Δ*S*) of the green mango slices at drying temperatures of 60°C, 65°C, and 70°C.

**TABLE 3:** Effect of pretreatments and drying temperatures on drying and thermodynamic characteristics.

Sample	Temp (°C)	*k* (h^−1^)	*D*_eff_ × 10^−10^ (m^2^s^−1^)	*R* ^2^	*E*_a_ (kJmol^−1^)	Δ*H (kJmol^−1^)*	Δ*G (kJmol^−1^)*	Δ*S (J mol⁻¹ K⁻¹)*
Control	60	0.593	1.04	0.9959	28.67	25.91	142.84	−350.99
	65	0.676	1.19	0.9956		25.86	144.59	−351.11
	70	0.802	1.41	0.9922		25.82	146.35	−351.23
CA	60	0.614	1.08	0.9876	26.99	24.22	142.74	−355.77
	65	0.684	1.20	0.9761		24.18	144.52	−355.89
	70	0.816	1.43	0.9725		24.14	146.30	−356.02
HWB	60	0.758	1.33	0.995	39.72	36.97	142.20	−315.86
	65	0.896	1.58	0.9932		36.93	143.78	−315.98
	70	1.152	2.03	0.993		36.89	145.36	−316.11
SB	60	0.635	1.12	0.8543	21.82	19.05	142.65	−371.01
	65	0.699	1.23	0.9019		19.00	144.50	−371.14
	70	0.799	1.41	0.9182		18.96	146.36	−371.26

Abbreviations: Δ*G*, Gibbs free energy; Δ*H*, enthalpy change; ΔS, entropy of activation; CA, citric acid; *D*_eff_, moisture diffusivity; *E*_a_, activation energy; HWB, hot water blanched; *k*, drying rate constant; *R* ^2^, coefficient of determination; SB, steam blanched; Temp, temperature.

**FIGURE 5:**
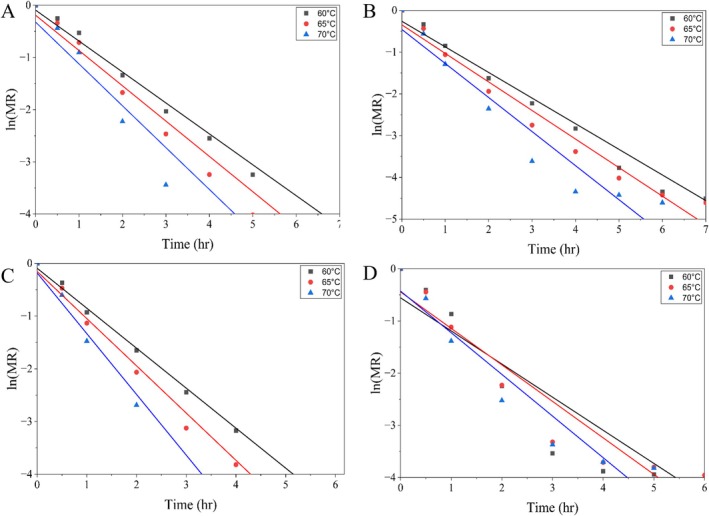
Drying curves of green mango slices at different drying temperature levels (60°C, 65°C, and 70°C) showing ln (MR) against drying time (h) for control (A), 1% citric acid treated (B), hot water blanched (C), and steam blanched (D) samples.

The drying rate constant (*k*) represents how quickly moisture leaves the fruit during the drying process, with higher values indicating faster water loss. In this study, k increased steadily with temperature across all treatments, ranging from 0.593 to 1.152 h^−1^, suggesting faster drying process of green mango samples. Among the pretreatments, HWB produced the highest *k* values (0.758–1.152 h^−1^), followed by CA and SB, even though SB showed slightly higher *k* values than CA until the drying temperature was 65°C. This shift suggests that the acid treatment became more effective at higher temperatures due to its impact on pectin structure and cell wall permeability (de Moura et al. 2024; Yao et al. 2020). On the other hand, the untreated samples were constantly the slowest to dry. Most treatments exhibited *R* ^2^ values above 0.90, indicating a good agreement between the experimental data and the predicted model (Table 3). However, a comparatively lower *R* ^2^ value was observed for the SB‐treated sample at 60°C (*R* ^2^ = 0.8543). As shown in Figure 5, the experimental points at the early stage of drying deviate slightly from the linear trend. This behavior may be related to structural changes caused by steam blanching, which can alter cell integrity and lead to uneven moisture redistribution at lower drying temperatures. Although the Modified Midilli I model provided excellent empirical fitting of the drying curves (*R* ^2^ > 0.99), *D*_eff_ is based on simplified Fickian diffusion assumptions, including uniform internal moisture migration, constant diffusivity, and negligible structural changes during drying. These assumptions may not be fully satisfied in SB due to heterogeneous moisture transport behavior during the initial drying stage. Therefore, empirical thin‐layer models may accurately describe the overall drying behavior even when diffusivity‐based linear fitting exhibits comparatively lower *R* ^2^ values.

*D*_eff_ represents the rate of water transfer during drying, where higher *D*_eff_ values indicate greater moisture mobility and stronger dehydration capability (Wang et al. 2020). For the drying of food materials, the effective moisture diffusivity typically ranges from 10^−12^ to 10^−6^ m^2^ s^−1^ (Tripathy and Srivastav 2024). As shown in the table, *D*_eff_ increased with increasing drying air temperature across all treatments, with HWB recording the highest diffusivity values (up to 2.03 × 10^−10^ m^2^ s^−1^) among all pretreatments. The enhanced diffusivity observed for HWB suggests that water blanching promoted structural softening and increased cell permeability, thereby reducing internal resistance to moisture migration. Direct contact between water and the mango tissue may also have improved heat transfer efficiency, causing faster moisture removal and shorter drying duration. In contrast, SB likely resulted in comparatively less uniform thermal penetration, whereas CA pretreatment mainly influenced enzymatic activity and surface chemistry rather than substantially enhancing internal moisture transport. Similar pretreatment‐induced enhancement of moisture diffusivity during air drying has also been reported for mango (Mishra et al. 2021), apricots (I. Doymaz 2004; Pala et al. 1996), pepper (Geng et al. 2022), white yam (Ajah et al. 2023), and cassava tubers (Nainggolan et al. 2023). Overall, the combined effects of elevated temperature and pretreatment contributed to improved heat and mass transfer, thereby reducing duration and enhancing drying efficiency. The relationship between *D*_eff_ and temperature for different samples can be expressed by the following equations (Putranto and Chen 2016).

(28) $$\text{Control}:D_{\mathrm{eff}}=3.25E-6\;\exp\;\left(\frac{-28675}{\mathrm{RT}}\right)$$

(29) $$1\%\text{citric acid}:D_{\mathrm{eff}}=1.83E-6\;\exp\;\left(\frac{-26999}{\mathrm{RT}}\right)$$

(30) $$\mathrm{Hot}\;\text{water blanched}:D_{\mathrm{eff}}=2.22E-4\;\exp\;\left(\frac{-39717}{\mathrm{RT}}\right)$$

(31) $$\text{Steam blanched}:D_{\mathrm{eff}}=2.92E-7\;\exp\;\left(\frac{-21816}{\mathrm{RT}}\right)$$

**FIGURE 6:**
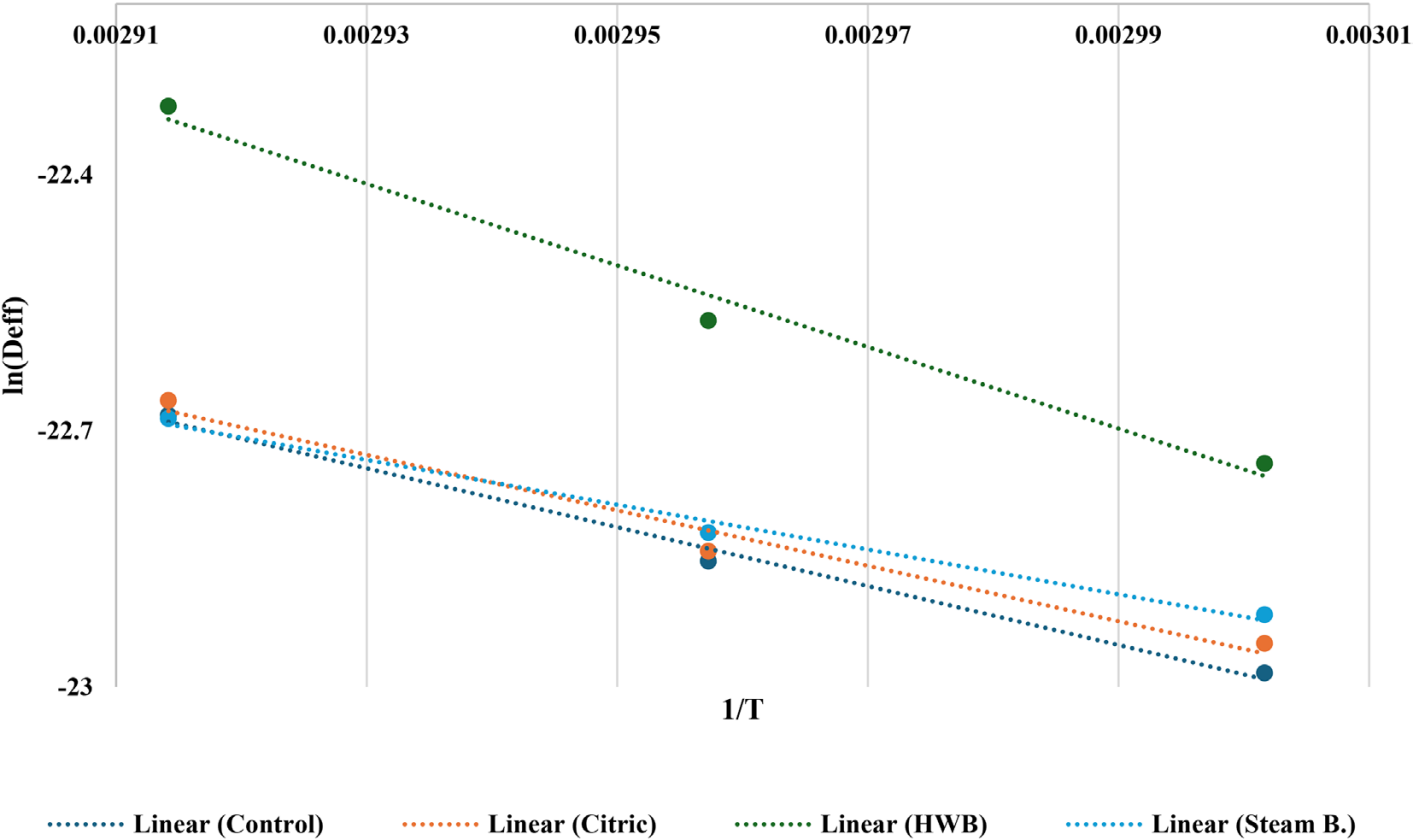
ln (*D*_eff_) versus absolute temperature (1/*T*) of different pretreatments showing Arrhenius type relationship between effective diffusivity and temperature to determine activation energy.

The relationship between ln (*D*_eff_) and the reciprocal of absolute temperature (1/*T*) was evaluated using linear regression, and *R* ^2^ was used to assess the goodness‐of‐fit of the Arrhenius model (Figure 6). A strong linear relationship was observed between ln (*D*_eff_) and 1/*T* for all pretreatments, with *R* ^2^ values of 0.9928, 0.9723, 0.9848, and 0.9894 for the control, CA, HWB, and SB treatments respectively, confirming that the temperature dependence of moisture diffusivity follows an Arrhenius‐type behavior.

Moreover, *E*_a_ represents the minimum energy required to initiate moisture diffusion during the drying process (Table 3). In this study, SB‐treated samples showed the lowest *E*_a_ (21.82 kJ mol^−1^), whereas HWB samples displayed the highest *E*_a_ (39.72 kJ mol^−1^). Although HWB samples exhibited faster drying and higher *D*_eff_, the higher *E*_a_ indicates that moisture diffusion in these samples was more sensitive to temperature changes. The structural disruption caused by HWB likely increased internal permeability and moisture mobility. However, the increase in diffusivity with temperature was more pronounced compared to other treatments, resulting in a steeper Arrhenius slope (ln *D*_eff_ vs. 1/*T*) and consequently a higher apparent *E*_a_. Therefore, the higher *E*_a_ observed for HWB reflects greater sensitivity of moisture diffusion to temperature changes rather than increased resistance to mass transfer (Kaveh et al. 2020). In contrast, SB introduces less severe tissue breakdown, causing more uniform diffusion paths with lower temperature sensitivity.

The values obtained in this study fall within the typical range for food materials (12–110 kJmol^−1^) (Putranto and Chen 2016) as well as aligns the range typically observed in food drying processes, such as piquin pepper (Ovando‐Medina 2023), lemon basil leaves (Mbegbu et al. 2021), potatoes (Singh and Talukdar 2019), orange (Bozkir 2020), and yam slices (Ojediran et al. 2020).

The associated thermodynamic parameters provided additional insight into the nature of the drying process. The enthalpy change (Δ*H*) confirms that dehydration is an endothermic process (Δ*H* > 0). For Δ*G*, the value increased from around 142 to 146 kJ mol⁻¹ across treatments, suggesting that moisture removal remained nonspontaneous throughout the process (Qiu et al. 2018). Δ*S* determines the matter chaos, energy change, and process stability. Interestingly, we found Δ*S* decreasing with increasing temperature, which indicates a more ordered transition state during moisture removal, suggesting that the drying process involves structured molecular rearrangements rather than random diffusion (Georgieva et al. 2012). These findings are consistent with previous literature reported on acerola (Mercali et al. 2013) and mulberry (Zhou et al. 2017) drying processes.

### 3.4 Energy Efficiency and Specific Energy Consumption

Table 4 summarizes the energy performance indicators, including thermal efficiency (η), SEC, MER, and SMER. In our study, both drying temperature and pretreatment had a strong influence on the energy parameters. Across all samples, the energy efficiency increased as temperature increased from 60°C to 70°C. This increase in efficiency at higher temperatures can be explained by more effective heat transfer and faster internal moisture movement. Elevated drying temperatures increase vapor pressure gradients and diffusion rates, accelerating moisture removal and reducing drying time. Therefore, less energy is required to evaporate each unit of water. These results align with previous studies where researchers found improved energy efficiency at higher drying temperatures (Abbaspour‐Gilandeh et al. 2021; Yamchi et al. 2024).

**TABLE 4:** Effect of pretreatments and drying temperatures on energy consumption and efficiency.

Pretreatment	Temp (°C)	*η* (%)	SEC (kWh kg^−1^ water)	MER (kg h^−1^)	SMER (kg kWh^−1^)
Control	60	9.4	6.96	0.0718	0.1436
	65	11.6	5.62	0.089	0.178
	70	14.7	4.42	0.1133	0.2265
CA	60	12.2	5.37	0.0932	0.1863
	65	13	5.01	0.0999	0.1998
	70	16.5	3.93	0.1272	0.2544
HWB	60	14.5	4.53	0.1104	0.2209
	65	19.4	3.37	0.1485	0.2969
	70	25.4	2.56	0.1953	0.3905
SB	60	12.85	5.09	0.0982	0.1964
	65	12.9	5.05	0.0989	0.1979
	70	19.95	3.26	0.1536	0.3072

Abbreviations: *η*, energy efficiency; CA, citric acid; HWB, hot water blanched; MER, moisture extraction rate; SB, steam blanched; SEC, specific energy consumption; SMER, specific moisture extraction rate; Temp, temperature.

Pretreatments also exhibited a clear and significant impact on energy efficiency and other energy‐related parameters. All pretreated samples performed better than untreated ones, with higher *η* and MER, along with lower SEC values. At 70°C, HWB achieved the highest η (25.4%), SMER (0.3905 kg kWh^−1^) and the lowest SEC (2.56 kWh kg^−1^). This improvement can be attributed to the blanching process, which disrupts cellular structures and enhances internal moisture mobility for faster evaporation with less energy input (Adeyi et al. 2022).

### 3.5 Techno‐Functional Properties and Total Phenolic Content

#### 3.5.1 Bulk Density, Water and Oil Absorption Capacity

Table 5 shows the influence of different pretreatments on the techno‐functional properties of the dried mango powders. A two‐way ANOVA with replicates confirmed significant differences in techno‐functional properties across pretreatments and temperatures (*p* < 0.05). Detailed ANOVA results are provided in Table S6. For each sample group, the value represents the mean ± standard deviation of the dried mango powder.

**TABLE 5:** Functional properties of dried green mango powder.

TF property	Temp (°C)	Control	CA	HWB	SB
Bulk density	60	0.65 ± 0.04^Aa^	0.73 ± 0.05^Aa^	0.72 ± 0.05^Aa^	0.77 ± 0.11^Aa^
	65	0.71 ± 0.08^Aa^	0.75 ± 0.06^Aa^	0.77 ± 0.07^Aa^	0.78 ± 0.03^Aa^
	70	0.75 ± 0.04^Aab^	0.71 ± 0.09^Ac^	0.81 ± 0.03^Aab^	0.86 ± 0.05^Aa^
WAC	60	0.50 ± 0.05^Cb^	0.54 ± 0.06^Bb^	0.75 ± 0.05^Ca^	0.77 ± 0.06^Ba^
	65	0.80 ± 0.04^Bc^	0.62 ± 0.07^Bd^	1.21 ± 0.08^Ba^	0.98 ± 0.07^Ab^
	70	1.11 ± 0.07^Ab^	0.93 ± 0.06^Ac^	1.70 ± 0.07^Aa^	0.83 ± 0.05^ABc^
OAC	60	0.59 ± 0.05^Ca^	0.19 ± 0.10^Bb^	0.23 ± 0.06^Bb^	0.24 ± 0.06^Ab^
	65	0.73 ± 0.06^Ba^	0.38 ± 0.07^ABb^	0.33 ± 0.07^ABb^	0.18 ± 0.04^Ac^
	70	0.85 ± 0.04^Aa^	0.48 ± 0.09^Ab^	0.49 ± 0.09^Ab^	0.27 ± 0.10^Ac^

*Note:* Values expressed are mean ± standard deviation. Superscript with different capital letters within the same column indicate significant differences among temperatures for a given pretreatment (*p* < 0.05). Different lowercase letters within the same row indicate significant differences among pretreatments at the same temperature (*p* < 0.05).

Abbreviations: CA, citric acid; HWB, hot water blanched; OAC, oil absorption capacity; SB, steam blanched; WAC, water absorption capacity.

Bulking properties reflect the flowability of powder, which depends on the interaction between the powdered particles. These interactions are dependent on several factors including moisture content, particle size distribution, pore space, and the compactness of the particles. In our study, bulk density of the dried green mango powder ranged from 0.65 to 0.86 g cm^−3^ depending on drying temperature and pretreatment, which falls within the usual range for dried powders (Jamalullail et al. 2022; Mishra et al. 2021; Sogi et al. 2013). However, statistical analysis showed that temperature did not significantly affect bulk density within each pretreatment group, as indicated by the identical capital letters in each column (*p <* 0.05). Similarly, differences among pretreatments at the same temperature were generally not significant, except for minor variations observed at 70°C, where SB exhibited the highest bulk density. These results suggest that both drying temperature and pretreatment had limited influence on the bulk density of green mango powder.

WAC evaluates the water holding capacity of dried powder. W ranged from 0.50 to 1.70 g water g^−1^ powder and was strongly influenced by both drying temperature and pretreatment. In most cases, WAC increased with increasing drying temperature, with HWB‐treated powder exhibiting the highest value at 70°C. Pretreatment also had a significant effect on WAC. At all temperatures, blanched samples consistently exhibited higher WAC values, indicating increased hydrophilicity. This enhancement is likely associated with the exposure of polar functional groups resulting from thermal disruption of cell wall structures. Blanching‐induced structural alterations, including cell wall permeabilization and partial degradation of macromolecular networks, may increase the availability of water‐binding sites, thereby improving the overall water absorption capacity of the powder. These results are in line with the studies on mango kernel (0.92–1.63 g water g^−1^ powder) (Sogi et al. 2013), dried chickpeas (Farooq and Wani 2025), pears (0.31 and 1.66 g water/g powder) (Du Toit et al. 2019), and potato starch (0.78–1.16 g water g^−1^ powder) (Kumar et al. 2020).

OAC plays a key role in how well flavors are retained and how the product feels in the mouth, and therefore, we determined the OAC of dried mango powder across different pretreated samples. In our study, OAC varied between 0.18 and 0.85 g oil g^−1^ powder across different treatments. In contrast to WAC, the control samples consistently exhibited the highest OAC values, whereas powders obtained from blanching treatments (HWB and SB) showed relatively lower OAC values, suggesting that thermal pretreatment might be attributed to changes in surface properties and a loss of hydrophobicity of the ground samples (Kumar et al. 2020). This reduction in OAC with pretreatments is consistent with earlier studies on dried plum (Okpala and Gibson‐Umeh 2013) and mango kernel (Ojha et al. 2019).

#### 3.5.2 Total Phenolic Content

TPC of dried green mango powder varied significantly (*p* < 0.05) with both pretreatment type and processing temperature. Across all treatments, TPC ranged between 75.49 ± 5.20 and 143.83 ± 10.33 mg GAE/100 g DW (Table 6), which aligns with previously reported values (Muralidhara et al. 2019; Nguyen et al. 2025; Nyangena, Owino, Ambuko, and Imathiu 2019; Nyangena, Owino, Imathiu, and Ambuko 2019). A clear declining trend in TPC was observed as temperature increased from 60°C to 70°C across various samples, suggesting the thermal sensitivity of native phenolic compounds in fruit tissues. Similar outcomes have also been reported in previous studies (Fratianni et al. 2020; Nguyen et al. 2025). Pretreatments mitigated this loss to a certain extent. Samples treated with acidic solution exhibited higher TPC than the control at all temperatures, aligning with previous reports that organic acids can stabilize phenolic compounds by lowering pH and preventing enzymatic oxidation (Alfieri et al. 2020; Luan et al. 2023).

**TABLE 6:** Total phenolic content of pretreated dried powder at different drying temperatures.

Temperature (°C)	Control	CA	HWB	SB
60	103.07 ± 3.44^Ac^	124.92 ± 5.76^Ab^	138.42 ± 6.69^Aa^	143.83 ± 10.33^Aa^
65	91.40 ± 9.36^Ab^	117.24 ± 6.02^Aa^	121.60 ± 5.10^Ba^	134.83 ± 7.58^Aa^
70	75.49 ± 5.20^Bc^	93.94 ± 6.09^Bb^	112.50 ± 4.07^Ba^	116.53 ± 5.65^Ba^

*Note:* Superscript with different capital letters within the same column indicate significant differences among temperatures for a given pretreatment (*p* < 0.05). Different lowercase letters within the same row indicate significant differences among pretreatments at the same temperature (*p* < 0.05).

Abbreviations: CA, citric acid; HWB, hot water blanched; SB, steam blanched.

Moreover, blanching further improved TPC, with the highest retention/enhancement observed in SB samples at 60°C (143.83 ± 10.33 mg GAE/100 g DW). This improvement is likely due to effective inactivation of polyphenol oxidase (PPO) and peroxidase (POD) enzymes that are responsible for phenolic degradation during processing (Ijod et al. 2025; Ndiaye et al. 2009). SB generally produces faster heat penetration with reduced leaching as SB minimizes direct contact with water, which preserves water‐soluble phenolics more efficiently than HWB (Demczuk Junior 2017). Although SB resulted in higher phenolic retention, HWB demonstrated superior drying performance and energy efficiency due to enhanced moisture diffusivity and reduced drying time, indicating a trade‐off between phytochemical preservation and process efficiency.

### 3.6 Sensory Analysis

**FIGURE 7:**
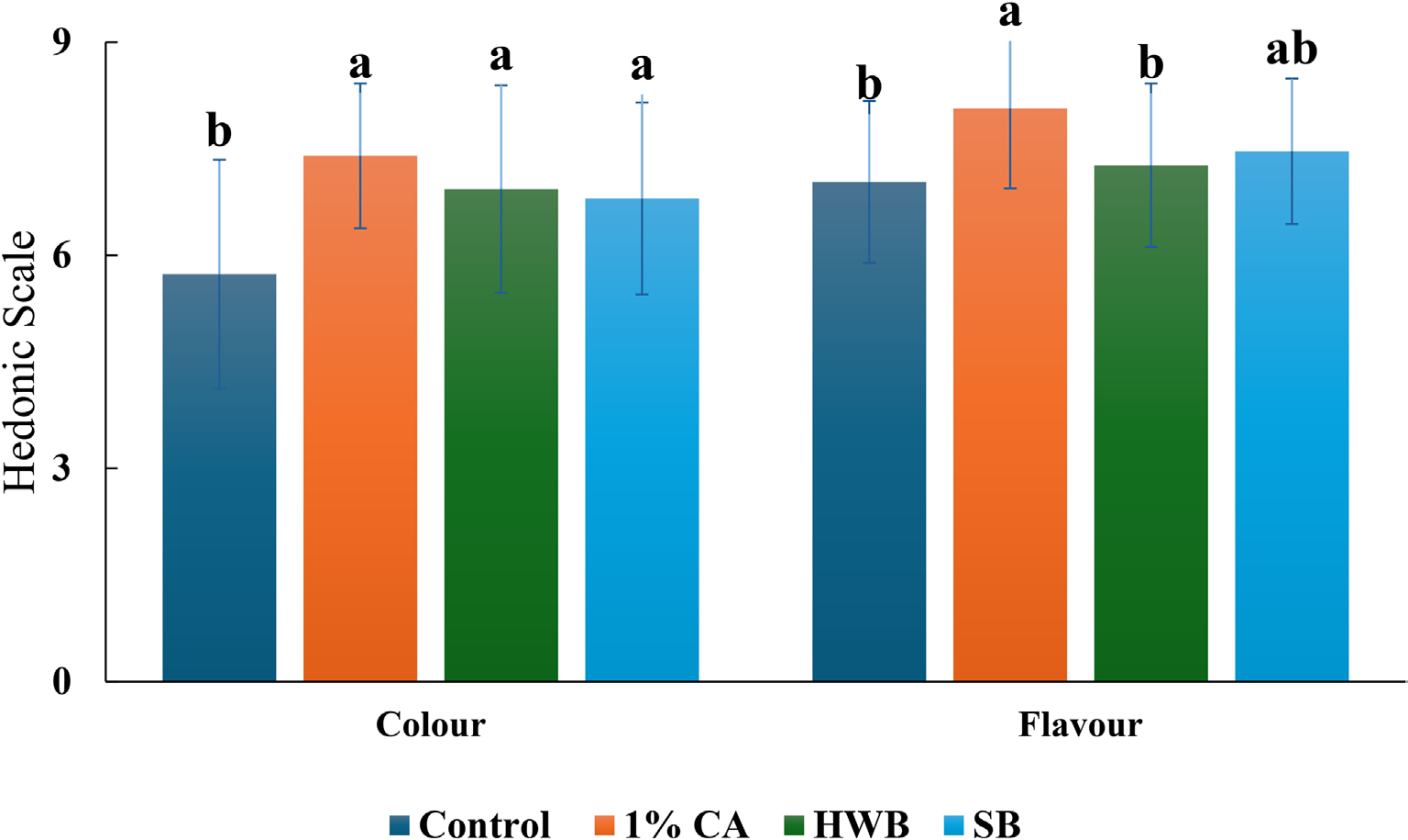
Organoleptic evaluation of color and flavor of dried green mango powder. Data are presented as mean with error bars showing the standard deviation. Bars with different letters represent significantly different at 5% level of significance. CA, citric acid; HWB, hot water blanching; SB, steam blanching.

Figure 7 presents the sensory scores for color and flavor of the mango powders prepared using different pretreatments. In terms of color, all the pretreated samples scored significantly better (*p* < 0.05) than the control samples (Table S6). Among the samples, CA‐treated samples obtained the highest mean score, which is consistent with the role of organic acids in slowing enzymatic browning and protecting natural pigments. This likely contributed to the powders' brighter appearance and the preservation of subtle fruity notes. The HWB and SB powders also received acceptable scores. Blanching briefly inactivates the enzymes that cause browning, so the color stays stable during drying.

For flavor, CA samples scored the highest mean among the samples, followed by SB, whereas HWB scored lower than the other pretreated samples. The heat treatment of blanching may cause a small loss of aroma compounds, which is likely why the flavor score of blanched samples was lower than the acidic treated samples. In contrast, control samples consistently scored the lowest (*p* < 0.05) scores among the samples. Without any pretreatment, the fruit is more prone to enzymatic and oxidative browning during drying, which promotes pigment degradation and the development of off flavors due to lipid oxidation and heat‐induced reactions (Boudboud et al. 2023), which leads to a darker color and a less vibrant flavor. Overall, the sensory results indicate that CA pretreatment provided the best balance of color and flavor, while blanched samples offered a strong alternative. These findings highlight the importance of pretreatment in maintaining the sensory quality of dried mango powders.

### 3.7 Life Cycle Assessment

A LCA was conducted to evaluate and compare the environmental performance of control and different pretreatment techniques used prior to processing based on the most efficient drying condition. Drying at 70°C was selected as the representative condition for the LCA because it showed better overall process efficiency compared with higher energy efficiency (*η*) and SEC within the studied range. Since electricity consumption during drying was identified as the dominant contributor to environmental impacts, the most energy‐efficient condition was considered the most appropriate basis for comparing the environmental performance of different pretreatment methods. As the treated samples showed similar physicochemical characteristics, the focus was placed on identifying the treatment with the lowest environmental impact. The LCA was conducted based on ReCiPe 2016 MidPoint (H) methodology. The quantitative results for the control, CA, HWB, and SB samples are presented in Table 7.

**TABLE 7:** The environmental impacts of all categories calculated with ReCiPe MidPoint (H) for control and pretreated samples.

Impact category	Abbreviation	Unit	Control	CA	HWB	SB
Global warming	GW	kg CO_2_ eq	4.81	4.94	3.87	4.36
Stratospheric ozone depletion	SOP	kg CFC‐11 eq	8.16 × 10^−6^	8.42 × 10^−6^	7.72 × 10^−6^	7.95 × 10^−6^
Ionizing radiation	IR	kBq Co‐60 eq	7.11 × 10^−2^	7.59 × 10^−2^	6.73 × 10^−2^	6.95 × 10^−2^
Ozone formation, Human health	OF, HH	kg NOx eq	8.49 × 10^−3^	8.81 × 10^−3^	6.93 × 10^−3^	7.74 × 10^−3^
Fine particulate matter formation	FP	kg PM_2.5_ eq	7.00 × 10^−3^	7.30 × 10^−3^	6.24 × 10^−3^	6.64 × 10^−3^
Ozone formation, Terrestrial ecosystems	OF, TE	kg NOx eq	8.75 × 10^−3^	9.07 × 10^−3^	7.17 × 10^−3^	7.99 × 10^−3^
Terrestrial acidification	TA	kg SO_2_ eq	2.35 × 10^−2^	2.43 × 10^−2^	2.11 × 10^−2^	2.24 × 10^−2^
Freshwater eutrophication	FEu	kg P eq	8.24 × 10^−4^	8.81 × 10^−4^	7.81 × 10^−4^	8.05 × 10^−4^
Marine eutrophication	MEu	kg N eq	1.18 × 10^−3^	1.23 × 10^−3^	1.18 × 10^−3^	1.18 × 10^−3^
Terrestrial ecotoxicity	TE	kg 1,4‐DCB	7.59	8.03	6.35	6.98
Freshwater ecotoxicity	FEc	kg 1,4‐DCB	6.41 × 10^−2^	6.81 × 10^−2^	5.64 × 10^−2^	6.04 × 10^−2^
Marine ecotoxicity	MEc	kg 1,4‐DCB	8.68 × 10^−2^	9.24 × 10^−2^	7.61 × 10^−2^	8.16 × 10^−2^
Human carcinogenic toxicity	HCT	kg 1,4‐DCB	7.51 × 10^−2^	8.03 × 10^−2^	6.94 × 10^−2^	7.27 × 10^−2^
Human noncarcinogenic toxicity	HNT	kg 1,4‐DCB	1.51	1.63	1.40	1.46
Land use	L	m^2^a crop eq	7.87 × 10^−1^	8.20 × 10^−1^	7.86 × 10^−1^	7.87 × 10^−1^
Mineral resource scarcity	MRS	kg Cu eq	1.21 × 10^−2^	1.25 × 10^−2^	1.18 × 10^−2^	1.20 × 10^−2^
Fossil resource scarcity	FS	kg oil eq	1.39	1.42	1.04	1.24
Water consumption	WC	m^3^	1.22	1.22	1.21	1.21

Abbreviations: CA, citric acid; HWB, hot water blanched; SB, steam blanched.

The environmental impacts of the different pretreatment strategies varied considerably across the evaluated midpoint categories. Among the treatments, HWB generally exhibited lower environmental impacts compared with the control, CA, and SB samples in most categories. In particular, HWB showed lower global warming potential (3.87 kg CO_2_ eq), terrestrial acidification (2.11 × 10^−2^ kg SO_2_ eq), and fine particulate matter formation (6.24 × 10^−3^ kg PM_2.5_ eq), resulting in reduced greenhouse gas emissions and lower atmospheric pollutant generation. Lower impacts were also observed for several toxicity‐related categories, including terrestrial ecotoxicity (6.35 kg 1,4‐DCB eq), freshwater ecotoxicity (5.64 × 10^−2^ kg 1,4‐DCB eq), and human carcinogenic toxicity (6.94 × 10^−2^ kg 1,4‐DCB eq). This suggests comparatively lower emission‐related environmental burdens associated with the HWB treatment. The comparatively improved environmental performance of HWB is likely associated with its shorter drying duration and lower energy requirement, which reduced electricity consumption during the drying process. Since electricity use was identified as the dominant contributor to most impact categories, improvements in drying efficiency directly influenced the overall environmental profile.

In contrast, CA exhibited the highest impacts across most categories, including stratospheric ozone depletion (8.42 × 10^−6^ kg CFC‐11 eq) and mineral resource scarcity (1.25 × 10^−2^ kg Cu eq), possibly due to the additional material and processing requirements associated with CA treatment. SB exhibited intermediate environmental performance, generally showing lower impacts than the control and CA but higher impacts than HWB. Interestingly, the control samples showed slightly lower impacts in the marine eutrophication category, as no additional pretreatment inputs or associated wastewater streams were generated prior to drying.

Overall, the findings suggest that pretreatments capable of improving drying efficiency may contribute to reduced environmental burdens during green mango processing. Similar observations have been reported in previous studies, where improved drying kinetics and reduced energy consumption contributed to lower environmental impacts in food processing systems. However, these findings should be interpreted within the limitations of the gate‐to‐gate system boundary adopted in this study, which excluded broader upstream agricultural variability and downstream distribution, packaging, storage, and consumer‐use stages. Therefore, the environmental conclusions reflect relative process‐level performance under the evaluated laboratory‐scale conditions rather than full cradle‐to‐grave sustainability.

#### 3.7.1 Impact Assessment of Different Pretreated Drying Samples

**FIGURE 8:**
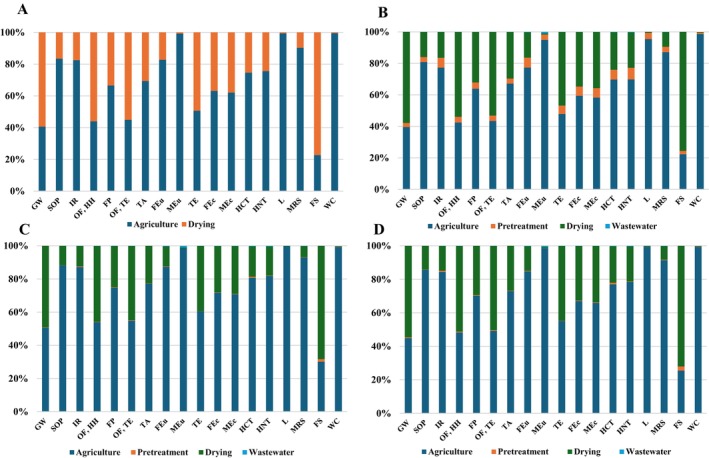
Individual impact assessment of control (A), 1% citric acid treated (B), hot water blanched (C), and steam blanched (D) dried mango slices. FEc, freshwater ecotoxicity; FEu, freshwater eutrophication; FP, fine particulate matter formation; FS, fossil resource scarcity; GW, global warming; HCT, human carcinogenic toxicity; HNT, human non‐carcinogenic toxicity; IR, ionizing radiation; L, land use; MEc, marine ecotoxicity; MEu, marine eutrophication; MRS, mineral resource scarcity; OF, HH, ozone formation (human health); OF, TE, ozone formation (terrestrial ecosystems); SOP, stratospheric ozone depletion; TA, terrestrial acidification; TE, terrestrial ecotoxicity; WC, water consumption.

It is evident that the control and CA generated higher emissions across nearly all midpoint impact categories compared to the HWB and SB treatments. To better illustrate the variations, Figure 8A–D illustrates the relative contributions of different life cycle stages, including agriculture, pretreatment, drying, and wastewater management. Among these stages, agriculture and drying process were the main contributors to the overall environmental impact. The drying step contributed especially high impacts for both the control and CA samples, since their drying times were longer (6 h) compared to the HWB and SB processes (4 and 5 h, respectively). As electricity consumption during drying was the dominant contributor within the defined system boundary, longer processing times translated directly into higher cumulative energy use and associated emissions. Although lower drying temperatures may reduce thermal degradation of food materials, they significantly prolong drying time due to lower moisture diffusivity and weaker vapor pressure gradients. Because electricity demand in hot‐air drying systems is strongly dependent on operating time, extended drying at lower temperatures can increase total energy consumption and the resulting environmental burdens. Consequently, within the investigated range, drying at 70°C resulted in lower overall environmental impacts due to improved energy efficiency and shorter drying duration. Similarly, improved drying kinetics for the blanched samples further reduced electricity demand and associated emissions. In contrast, the CA samples showed comparatively higher impacts due to the additional material input and the upstream environmental burden associated with CA production and use.

Overall, the lower environmental footprint observed for HWB is possibly due to its improved process efficiency, including reduced drying time and lower SEC, and higher SMER. Therefore, within the conditions and boundaries considered in this study, HWB exhibited the lowest environmental impacts among the evaluated treatments. This finding highlights the importance of process efficiency and energy demand in determining the overall sustainability of hot‐air drying systems.

## 4 Limitations and Future Directions

Despite providing valuable insights into the drying behavior, energy performance, techno‐functional properties, and environmental impact of pretreated green mango slices, this study has several limitations. The drying experiments were conducted as single continuous runs for each treatment–temperature combination, which is common in thin‐layer drying studies but may limit statistical representation of biological variability across independent experimental batches. In addition, all mango samples were obtained from a single harvesting batch, which ensured experimental uniformity but may restrict the applicability of the results across different cultivars, maturity stages, and growing conditions. Therefore, the kinetic observations should be interpreted as process‐specific trends rather than estimates of batch‐to‐batch variability. Future studies should include replicated drying runs using multiple harvesting batches to improve the robustness and generalizability of the findings. Moreover, the pH of mango slices after CA pretreatment was not directly measured. Since pH strongly influences enzymatic browning inhibition and phenolic stability, future studies should quantify post‐treatment pH to better explain the relationship between acidification and quality retention during drying. The air velocity of the drying system was factory‐set and could not be adjusted, limiting evaluation of airflow effects on drying kinetics.

Another limitation relates to the scope of quality and sustainability assessments. The study primarily evaluated TPC as a representative bioactive compound indicator, while other antioxidant activity measurements (e.g., DPPH, ABTS, or FRAP) were not included. Similarly, the sensory evaluation focused only on color and flavor with a semi‐trained panel, without assessing additional attributes such as texture or overall acceptability. From an environmental perspective, the LCA was conducted under the most energy‐efficient drying temperature (70°C) to compare pretreatment strategies, rather than evaluating the full temperature range.

Future research should address these limitations by conducting replicated drying experiments across multiple batches of raw materials to improve statistical robustness. Investigating different mango cultivars, maturity levels, and harvesting conditions could also improve the generalizability of the findings. Further work should evaluate the effects of additional process parameters such as air velocity, slice thickness, and alternative pretreatment intensities, including optimization of CA concentration and treatment duration. Expanding the analysis of bioactive compounds by incorporating antioxidant activity assays and profiling of specific phenolic compounds would provide deeper insights into nutritional quality retention during drying. Moreover, future sustainability studies could conduct a comprehensive LCA across multiple drying temperatures and processing configurations. Finally, integrating advanced drying technologies (e.g., hybrid drying, infrared‐assisted drying, or heat pump drying) and performing techno‐economic analysis alongside LCA could further support the development of efficient and sustainable processing strategies for green mango valorization.

## 5 Conclusion

This study was initiated to dry green mango for minimizing the green mango waste and analyzed how chemical and physical pretreatments and drying temperatures influence the drying characteristics, energy consumption, functionality and environmental footprint. Higher temperatures naturally sped up drying, but the biggest improvements came from blanching, especially HWB, which consistently produced faster drying, higher moisture diffusivity, and better energy efficiency. Across all the thin‐layer models tested, the Midilli and Modified Midilli I models described the drying behavior most accurately. Pretreatments also showed impact on the functional, phytochemical and sensory properties of the powders, with HWB improving water absorption, SB retaining more phenolics at milder temperatures, and acidic treatment supporting color and flavor stability. LCA highlighted that HWB produced the lowest environmental impacts across most categories, while acidic treatment exhibited the highest burden due to upstream chemical production. Therefore, within the laboratory‐scale and gate‐to‐gate system boundary evaluated in this study, HWB offered the most favorable combination of drying efficiency, energy performance, and reduced environmental impacts, whereas CA pretreatment may be preferred when sensory quality is prioritized.

From an industrial perspective, the findings suggest that HWB prior to hot‐air drying may improve process efficiency by reducing drying time and energy demand while maintaining acceptable functional and sensory quality. These advantages could support the development of green mango powder as a value‐added ingredient for beverage mixes, seasoning formulations, instant soups, and functional food applications. The identified drying models may also assist in process optimization and scale‐up of industrial drying operations. Overall, the findings provide a robust foundation for the development of more sustainable and efficient drying processes for tropical fruit products.

## Author Contributions

**Md. Sajjad Hossain:** supervision, resources, project administration, writing – review and editing, conceptualization. **Mohammad Nurur Rahman:** conceptualization, supervision, resources, project administration. **Md Zahir Mahmud:** data curation. **S. M. Johir Rayhan:** conceptualization, methodology, investigation, writing – original draft. **S. K. Fahim Tahmid Boni:** conceptualization, software, visualization, writing – original draft, writing – review and editing, investigation, formal analysis. **Farzana Haque Reeti:** writing – original draft. **Pabitra Chandra Das:** supervision, writing – review and editing, conceptualization.

## Funding

The authors have nothing to report.

## Disclosure

The authors have nothing to report.

## Ethics Statement

The authors have nothing to report.

## Conflicts of Interest

The authors declare no conflicts of interest.

## Supporting information


**Table S1:** Life cycle inventory data for the process (per kg dried green mango).
**Table S2:** Drying constants and the drying coefficient of the selected thin layer drying models of control green mango slices.
**Table S3:** Drying constants and the drying coefficient of the selected thin layer drying models of 1% citric acid treated green mango slices.
**Table S4:** Drying constants and the drying coefficient of the selected thin layer drying models of hot water blanched green mango slices.
**Table S5:** Drying constants and the drying coefficient of the selected thin layer drying models of steam blanched green mango slices.
**Table S6:** ANOVA results for the effect of pretreatment and drying temperature on techno‐functional properties, total phenolic content, and organoleptic characteristics.
**Figure S1:** Comparison and validation of experimental moisture ratio and predicted moisture ratio using the best fitted drying model for control at different drying temperature: (A) 60°C, (B) 65°C, and (C) 70°C.
**Figure S2:** Comparison and validation of experimental moisture ratio and predicted moisture ratio using the best fitted drying model for 1% citric acid treated samples at different drying temperature: (A) 60°C, (B) 65°C, and (C) 70°C.
**Figure S3:** Comparison and validation of experimental moisture ratio and predicted moisture ratio using the best fitted drying model for hot water blanched samples at different drying temperature: (A) 60°C, (B) 65°C, and (C) 70°C.
**Figure S4:** Comparison and validation of experimental moisture ratio and predicted moisture ratio using the best fitted drying model for steam blanched samples at different drying temperature: (A) 60°C, (B) 65°C, and (C) 70°C.

## Data Availability

The data supporting the findings of this study are available within the article and its Supporting Information. Additional experimental data are available from the corresponding author upon reasonable request. Background inventory data used for the life cycle assessment were obtained from published literature and the Ecoinvent v3.9.1 database and are subject to the licensing restrictions of the respective sources. An earlier version of this work was posted as a preprint: “Effect of Pretreatments and Temperatures on the Drying Characteristics, Thermodynamic and Functional Properties of Oven‐Dried Green Mango Pulp ( *Mangifera indica* L.). https://doi.org/10.21203/rs.3.rs‐5500358/v1.”
