# Supplementary material for: Pretreatment‐Driven Modulation of Green Mango Drying: Kinetics, Energy Consumption, Techno‐Functionality, and Environmental Sustainability
Source: Food Sci Nutr. 2026 Aug 16;14(8):e72231. doi: 10.1002/fsn3.72231 (PMC13478560; doi:10.1002/fsn3.72231)
Supplement: Supplementary file 1 [file FSN3-14-e72231-s001.docx]

**Table S1.** Life cycle inventory data for the process (per kg dried green mango)

| **Agricultural Data per FU (1kg)** |  |  |  |  |  |  |
| --- | --- | --- | --- | --- | --- | --- |
| **Materials/fuels** | **Amount** | | **Unit** |  | **References** |  |
| Diesel | 0.0044 | | kg |  | (Ram & Verma, 2015) |  |
| Irrigation (Water) | 333 | | l |  | (Basset-Mens et al., 2018) |  |
| Phosphorous Fertilizer | 0.0067 | | kg |  | (Basset-Mens et al., 2018) |  |
| Potassium sulfate | 0.0208 | | kg |  | (Basset-Mens et al., 2018) |  |
| Nitrogen fertiliser | 0.015 | | kg |  | (Basset-Mens et al., 2018) |  |
| Pesticide, unspecified | 0.00417 | | kg |  | (Basset-Mens et al., 2018) |  |
| Land use change | 0.0000833 | | ha |  | (World Population Review, 2025) |  |
| **Drying data per FU (1kg)** |  | |  |  |  |  |
| **Input** | **Control** | **1% CA** | **HWB** | **SB** | **Unit** |  |
| Collected green mango | 3.6 | 3.6 | 3.6 | 3.6 | kg |  |
| Electricity, medium voltage (BD) | 4.08 | 4.08 | 2.72 | 3.4 | kWh |  |
| Citric acid | - | 0.02 | - | - | kg |  |
| Water | - | 1.98 | 2 | 1.5 | kg |  |
| Natural gas liquids | - | - | 0.017 | 0.034 | kg |  |
| **Output** |  |  |  |  |  |  |
| Wastewater | - | 2 | 1.8 | 0.3 | kg |  |
| Vapor | 2.6 | 2.6 | 3.4 | 3.8 | kg |  |

^†^CA= citric acid, HWB= hot water blanched, SB= steam blanched

**Table S2.** Drying constants and the drying coefficient of the selected thin layer drying models of control green mango slices.

| **Model** | **Temperature (ºC)** | **Drying constants**  **(k, k_1_)** | **Drying coefficients (n, a, b, c, g, h, α, β, δ)** | **SSE** | **R^2^** | **RMSE** | **χ^2^** |
| --- | --- | --- | --- | --- | --- | --- | --- |
| Newton | 60 | 0.6099 |  | 0.0060 | 0.9960 | 0.0245 | 0.0007 |
|  | 65 | 0.7596 |  | 0.0029 | 0.9977 | 0.0170 | 0.0003 |
|  | 70 | 0.9573 |  | 0.0034 | 0.9969 | 0.0207 | 0.0005 |
| Average | | | | 0.0041 | 0.9969 | 0.0207 | 0.0005 |
| Page | 60 | 0.5585 | n=1.1612 | 0.0016 | 0.9988 | 0.0128 | 0.0002 |
|  | 65 | 0.7315 | n=1.1223 | 0.0007 | 0.9995 | 0.0087 | 0.0001 |
|  | 70 | 0.9448 | n=1.1591 | 0.0009 | 0.9992 | 0.0105 | 0.0001 |
| Average | | | | 0.0011 | 0.9992 | 0.0107 | 0.0001 |
| Modified Page | 60 | 0.6055 | n=1.1612 | 0.0016 | 0.9988 | 0.0128 | 0.0002 |
|  | 65 | 0.7568 | n=1.1223 | 0.0007 | 0.9995 | 0.0087 | 0.0001 |
|  | 70 | 0.9522 | n=1.1591 | 0.0009 | 0.9992 | 0.0105 | 0.0001 |
| Average | | | | 0.0011 | 0.9992 | 0.0107 | 0.0001 |
| Henderson and Pabis | 60 | 0.6288 | a=1.0282 | 0.0049 | 0.9960 | 0.0222 | 0.0006 |
|  | 65 | 0.7743 | a=1.0172 | 0.0024 | 0.9978 | 0.0164 | 0.0003 |
|  | 70 | 0.9717 | a=1.0143 | 0.0032 | 0.9968 | 0.0199 | 0.0005 |
| Average | | | | 0.0035 | 0.9969 | 0.0195 | 0.0005 |
| **Midilli** | **60** | **0.5604** | **n=1.1927, a=1.0000, b=0.0020** | **0.0011** | **0.9991** | **0.0104** | **0.0002** |
|  | **65** | **0.7363** | **n=1.1499, a=0.9992, b=0.0020** | **0.0003** | **0.9997** | **0.0057** | **0.0001** |
|  | **70** | **0.9507** | **n=1.1830, a=0.9974, b=0.0019** | **0.0006** | **0.9994** | **0.0088** | **0.0002** |
| **Average** | | | | **0.0007** | **0.9994** | **0.0083** | **0.0001** |
| Modified Midilli I | 60 | 0.5680 | n=1.2104, a=0.9835, b=0.0157 | 0.0010 | 0.9992 | 0.0099 | 0.0002 |
|  | 65 | 0.7469 | n=1.1624, a=0.9863, b=0.0126 | 0.0003 | 0.9997 | 0.0055 | 0.0001 |
|  | 70 | 0.9606 | n=1.1893, a=0.9888, b=0.0086 | 0.0007 | 0.9993 | 0.0092 | 0.0002 |
| Average | | | | 0.0006 | 0.9994 | 0.0082 | 0.0001 |
| Modified Midilli II | 60 | 0.5604 | n=1.1927, b=0.0020 | 0.0011 | 0.9991 | 0.0104 | 0.0002 |
|  | 65 | 0.7372 | n=1.1488, b=0.0020 | 0.0003 | 0.9997 | 0.0057 | 0.0000 |
|  | 70 | 0.9538 | n=1.1794, b=0.0019 | 0.0006 | 0.9994 | 0.0088 | 0.0001 |
| Average | | | | 0.0007 | 0.9994 | 0.0083 | 0.0001 |
| Logarithmic | 60 | 0.6141 | a=1.0340, c=−0.0083 | 0.0047 | 0.9960 | 0.0217 | 0.0007 |
|  | 65 | 0.7726 | a=1.0175, c=−0.0006 | 0.0025 | 0.9978 | 0.0159 | 0.0004 |
|  | 70 | 0.9524 | a=1.0203, c=−0.0076 | 0.0030 | 0.9967 | 0.0174 | 0.0005 |
| Average | | | | 0.0034 | 0.9968 | 0.0183 | 0.0005 |
| Wang and Singh | 60 |  | a=−0.3691, b=0.0321 | 0.0492 | 0.9686 | 0.0701 | 0.0061 |
|  | 65 |  | a=−0.4361, b=0.0441 | 0.0601 | 0.9606 | 0.0817 | 0.0086 |
|  | 70 |  | a=−0.5271, b=0.0631 | 0.0662 | 0.9528 | 0.0910 | 0.0110 |
| Average | | | | 0.0585 | 0.9607 | 0.0809 | 0.0086 |
| Weibullian | 60 |  | α=1.6515, β=1.1612 | 0.0016 | 0.9988 | 0.0128 | 0.0002 |
|  | 65 |  | α=1.3213, β=1.1223 | 0.0007 | 0.9995 | 0.0087 | 0.0001 |
|  | 70 |  | α=1.0502, β=1.1591 | 0.0009 | 0.9992 | 0.0105 | 0.0001 |
| Average | | | | 0.0011 | 0.9992 | 0.0107 | 0.0001 |
| Weibullian I | 60 |  | n=1.1612, $\delta$=3.3871 | 0.0016 | 0.9988 | 0.0128 | 0.0002 |
|  | 65 |  | n=1.1223, $\delta$=2.7781 | 0.0007 | 0.9995 | 0.0087 | 0.0001 |
|  | 70 |  | n=1.1591, $\delta$=2.1567 | 0.0009 | 0.9992 | 0.0105 | 0.0001 |
| Average | | | | 0.0011 | 0.9992 | 0.0107 | 0.0001 |
| Peleg | 60 |  | a=1.3340, b=0.7941 | 0.0200 | 0.9840 | 0.0447 | 0.0025 |
|  | 65 |  | a=1.0292, b=0.8157 | 0.0154 | 0.9860 | 0.0413 | 0.0022 |
|  | 70 |  | a=0.7990, b=0.8266 | 0.0163 | 0.9835 | 0.0451 | 0.0027 |
| Average | | | | 0.0172 | 0.9845 | 0.0437 | 0.0025 |
| Silva and others | 60 |  | a=0.7885, b=−0.2203 | 0.0015 | 0.9989 | 0.0121 | 0.0002 |
|  | 65 |  | a=0.9379, b=−0.1966 | 0.0007 | 0.9995 | 0.0085 | 0.0001 |
|  | 70 |  | a=1.2336, b=−0.2750 | 0.0012 | 0.9988 | 0.0121 | 0.0002 |
| Average | | | | 0.0011 | 0.9991 | 0.0109 | 0.0002 |
| Cubic | 60 |  | a=−0.0002, b=0.0285, c=−0.3234, d=0.9121 | 0.0356 | 0.9704 | 0.0596 | 0.0059 |
|  | 65 |  | a=−0.0081, b=0.1228, c=−0.6038, d=0.9889 | 0.0021 | 0.9980 | 0.0154 | 0.0004 |
|  | 70 |  | a=−0.0141, b=0.1808, c=−0.7415, d=0.9864 | 0.0017 | 0.9983 | 0.0145 | 0.0004 |
| Average | | | | 0.0131 | 0.9889 | 0.0299 | 0.0023 |
| Demir et al. | 60 | 0.6214 | n=0.9883, a=1.0340, b=−0.0083 | 0.0047 | 0.9960 | 0.0217 | 0.0008 |
|  | 65 | 0.7694 | n=0.9940, a=1.0204, b=−0.0044 | 0.0023 | 0.9978 | 0.0162 | 0.0005 |
|  | 70 | 0.9623 | n=0.9897, a=1.0203, b=−0.0076 | 0.0030 | 0.9969 | 0.0194 | 0.0008 |
| Average | | | | 0.0034 | 0.9969 | 0.0191 | 0.0007 |
| Binomil | 60 |  | a=0.0283, b=−0.3311, c=0.9233 | 0.0368 | 0.9691 | 0.0606 | 0.0053 |
|  | 65 |  | a=0.0377, b=−0.3802, c=0.9017 | 0.0411 | 0.9613 | 0.0676 | 0.0069 |
|  | 70 |  | a=0.0535, b=−0.4551, c=0.8918 | 0.0451 | 0.9536 | 0.0751 | 0.0090 |
| Average | | | | 0.0410 | 0.9613 | 0.0678 | 0.0070 |
| Two-term | 60 | k=0.7367, k_1_=2.4717 | a=1.2352, b=−0.2363 | 0.0012 | 0.9991 | 0.0110 | 0.0002 |
|  | 65 | k=0.8983, k_1_=2.5475 | a=1.2216, b=−0.2221 | 0.0006 | 0.9996 | 0.0078 | 0.0001 |
|  | 70 | k=1.4000, k_1_=1.7743 | a=2.8638, b=−1.8654 | 0.0008 | 0.9993 | 0.0100 | 0.0002 |
| Average | | | | 0.0009 | 0.9993 | 0.0096 | 0.0002 |
| Hii and others | 60 | k=0.8941, k_1_=2.1642 | n=0.9062, a=1.5470, b=−0.5482 | 0.0012 | 0.9991 | 0.0107 | 0.0002 |
|  | 65 | k=1.0658, k_1_=2.3033 | n=0.9141, a=1.5498, b=−0.5504 | 0.0005 | 0.9996 | 0.0076 | 0.0001 |
|  | 70 | k=1.2997, k_1_=1.5873 | n=1.0516, a=2.7398, b=−1.7414 | 0.0008 | 0.9993 | 0.0099 | 0.0003 |
| Average | | | | 0.0008 | 0.9993 | 0.0094 | 0.0002 |

**Table S3.** Drying constants and the drying coefficient of the selected thin layer drying models of 1% citric acid treated green mango slices.

| **Model** | **Temperature (ºC)** | **Drying constants**  **(k, k_1_)** | **Drying coefficients (n, a, b, c, g, h, α, β, δ)** | **SSE** | **R^2^** | **RMSE** | **χ^2^** |
| --- | --- | --- | --- | --- | --- | --- | --- |
| Newton | 60 | 0.7797 |  | 0.0028 | 0.9974 | 0.0177 | 0.0004 |
|  | 65 | 0.9667 |  | 0.0026 | 0.9977 | 0.0170 | 0.0003 |
|  | 70 | 1.2008 |  | 0.0014 | 0.9986 | 0.0132 | 0.0002 |
| Average | | | | 0.0023 | 0.9979 | 0.0160 | 0.0003 |
| Page | 60 | 0.7755 | n=1.0199 | 0.0028 | 0.9975 | 0.0175 | 0.0004 |
|  | 65 | 0.9658 | n=1.0232 | 0.0025 | 0.9978 | 0.0168 | 0.0004 |
|  | 70 | 1.2099 | n=1.0553 | 0.0012 | 0.9990 | 0.0121 | 0.0002 |
| Average | | | | 0.0022 | 0.9981 | 0.0155 | 0.0003 |
| Modified Page | 60 | 0.7793 | n=1.0199 | 0.0028 | 0.9975 | 0.0175 | 0.0004 |
|  | 65 | 0.9665 | n=1.0232 | 0.0025 | 0.9978 | 0.0168 | 0.0004 |
|  | 70 | 1.1979 | n=1.0553 | 0.0012 | 0.9990 | 0.0121 | 0.0002 |
| Average | | | | 0.0022 | 0.9981 | 0.0155 | 0.0003 |
| Henderson and Pabis | 60 | 0.7876 | a=1.0087 | 0.0027 | 0.9975 | 0.0174 | 0.0004 |
|  | 65 | 0.9739 | a=1.0066 | 0.0026 | 0.9977 | 0.0168 | 0.0004 |
|  | 70 | 1.2075 | a=1.0054 | 0.0014 | 0.9986 | 0.0130 | 0.0002 |
| Average | | | | 0.0022 | 0.9979 | 0.0158 | 0.0003 |
| Midilli | 60 | 0.7896 | n=1.0447, a=1.0059, b=0.0021 | 0.0023 | 0.9977 | 0.0160 | 0.0005 |
|  | 65 | 0.9850 | n=1.0559, a=1.0040, b=0.0025 | 0.0019 | 0.9981 | 0.0143 | 0.0004 |
|  | 70 | 1.2308 | n=1.0830, a=1.0017, b=0.0023 | 0.0008 | 0.9992 | 0.0098 | 0.0002 |
| Average | | | | 0.0016 | 0.9984 | 0.0134 | 0.0003 |
| **Modified Midilli I** | **60** | **0.8079** | **n=1.0676, a=0.9883, b=0.0171** | **0.0021** | **0.9979** | **0.0154** | **0.0004** |
|  | **65** | **1.0160** | **n=1.0885, a=0.9855, b=0.0180** | **0.0016** | **0.9984** | **0.0133** | **0.0003** |
|  | **70** | **1.2637** | **n=1.1082, a=0.9882, b=0.0132** | **0.0007** | **0.9993** | **0.0091** | **0.0002** |
| **Average** | | | | **0.0015** | **0.9985** | **0.0126** | **0.0003** |
| Modified Midilli II | 60 | 0.7829 | n=1.0516, b=0.0022 | 0.0024 | 0.9977 | 0.0162 | 0.0004 |
|  | 65 | 0.9804 | n=1.0601, b=0.0025 | 0.0019 | 0.9981 | 0.0144 | 0.0003 |
|  | 70 | 1.2289 | n=1.0847, b=0.0023 | 0.0008 | 0.9992 | 0.0098 | 0.0002 |
| Average | | | | 0.0017 | 0.9983 | 0.0135 | 0.0003 |
| Logarithmic | 60 | 0.8091 | a=1.0025, c=0.0090 | 0.0025 | 0.9976 | 0.0167 | 0.0004 |
|  | 65 | 1.0052 | a=0.9984, c=0.0110 | 0.0021 | 0.9979 | 0.0152 | 0.0003 |
|  | 70 | 1.2273 | a=1.0005, c=0.0060 | 0.0012 | 0.9987 | 0.0124 | 0.0002 |
| Average | | | | 0.0019 | 0.9980 | 0.0148 | 0.0003 |
| Wang and Singh | 60 |  | a=−0.4329, b=0.0436 | 0.0810 | 0.9461 | 0.0949 | 0.0116 |
|  | 65 |  | a=−0.4637, b=0.0485 | 0.1344 | 0.9137 | 0.1222 | 0.0192 |
|  | 70 |  | a=−0.5548, b=0.0683 | 0.1351 | 0.9051 | 0.1300 | 0.0225 |
| Average | | | | 0.1168 | 0.9216 | 0.1157 | 0.0178 |
| Weibullian | 60 |  | α=1.2832, β=1.0199 | 0.0028 | 0.9975 | 0.0175 | 0.0004 |
|  | 65 |  | α=1.0346, β=1.0232 | 0.0025 | 0.9978 | 0.0168 | 0.0004 |
|  | 70 |  | α=0.8348, β=1.0553 | 0.0206 | 0.9855 | 0.0507 | 0.0034 |
| Average | | | | 0.0086 | 0.9936 | 0.0283 | 0.0014 |
| Weibullian I | 60 |  | n=1.0199, δ=2.9068 | 0.0028 | 0.9975 | 0.0175 | 0.0004 |
|  | 65 |  | n=1.0232, δ=2.3376 | 0.0025 | 0.9978 | 0.0168 | 0.0004 |
|  | 70 |  | n=1.0553, δ=1.8400 | 0.0012 | 0.9990 | 0.0121 | 0.0002 |
| Average | | | | 0.0022 | 0.9981 | 0.0155 | 0.0003 |
| Peleg | 60 |  | a=0.9655, b=0.8356 | 0.0106 | 0.9900 | 0.0343 | 0.0015 |
|  | 65 |  | a=0.7327, b=0.8672 | 0.0123 | 0.9879 | 0.0369 | 0.0018 |
|  | 70 |  | a=0.5766, b=0.8735 | 0.0109 | 0.9882 | 0.0369 | 0.0018 |
| Average | | | | 0.0113 | 0.9887 | 0.0361 | 0.0017 |
| Silva and others | 60 |  | a=0.8493, b=−0.0753 | 0.0026 | 0.9978 | 0.0168 | 0.0004 |
|  | 65 |  | a=1.0690, b=−0.0997 | 0.0024 | 0.9982 | 0.0162 | 0.0003 |
|  | 70 |  | a=1.3907, b=−0.1711 | 0.0010 | 0.9992 | 0.0113 | 0.0002 |
| Average | | | | 0.0020 | 0.9984 | 0.0148 | 0.0003 |
| Cubic | 60 |  | a=−0.0088, b=0.1282, c=−0.6084, d=0.9773 | 0.0083 | 0.9919 | 0.0304 | 0.0017 |
|  | 65 |  | a=−0.0153, b=0.1882, c=−0.7432, d=0.9760 | 0.0077 | 0.9917 | 0.0310 | 0.0019 |
|  | 70 |  | a=−0.0181, b=0.2175, c=−0.8132, d=0.9560 | 0.0135 | 0.9852 | 0.0411 | 0.0034 |
| Average | | | | 0.0098 | 0.9896 | 0.0342 | 0.0023 |
| Demir et al. | 60 | 0.7981 | n=1.0137, a=1.0025, b=0.0090 | 0.0025 | 0.9976 | 0.0167 | 0.0005 |
|  | 65 | 0.9985 | n=1.0066, a=0.9984, b=0.0110 | 0.0021 | 0.9979 | 0.0152 | 0.0004 |
|  | 70 | 1.1155 | n=1.1002, a=1.0005, b=0.0060 | 0.0012 | 0.9987 | 0.0124 | 0.0003 |
| Average | | | | 0.0019 | 0.9980 | 0.0148 | 0.0004 |
| Binomil | 60 |  | a=0.0360, b=−0.3662, c=0.8828 | 0.0540 | 0.9472 | 0.0775 | 0.0090 |
|  | 65 |  | a=0.0503, b=−0.4329, c=0.8735 | 0.0586 | 0.9366 | 0.0856 | 0.0117 |
|  | 70 |  | a=0.0535, b=−0.4444, c=0.8341 | 0.0854 | 0.9062 | 0.1033 | 0.0171 |
| Average | | | | 0.0660 | 0.9300 | 0.0888 | 0.0126 |
| Two-term | 60 | k=0.7876, k_1_=0.7876 | a=4.3459, b=−3.3372 | 0.0027 | 0.9975 | 0.0174 | 0.0005 |
|  | 65 | k=0.9739, k_1_=0.9739 | a=4.3925, b=−3.3859 | 0.0026 | 0.9977 | 0.0168 | 0.0005 |
|  | 70 | k=1.5122, k_1_=1.6027 | a=5.1240, b=−4.1212 | 0.0013 | 0.9989 | 0.0127 | 0.0003 |
| Average | | | | 0.0022 | 0.9980 | 0.0156 | 0.0005 |
| Hii and others | 60 | k=0.7834, k_1_=0.7834 | n=1.0127, a=4.3415, b=−3.3345 | 0.0027 | 0.9975 | 0.0174 | 0.0007 |
|  | 65 | k=0.9709, k_1_=0.9707 | n=1.0186, a=4.3856, b=−3.3808 | 0.0025 | 0.9979 | 0.0167 | 0.0006 |
|  | 70 | k=1.3631, k_1_=1.5093 | n=1.0474, a=4.8888, b=−3.8905 | 0.0208 | 0.9850 | 0.0510 | 0.0069 |
| Average | | | | 0.0087 | 0.9935 | 0.0284 | 0.0027 |

**Table S4.** Drying constants and the drying coefficient of the selected thin layer drying models of hot water blanched green mango slices.

| **Model** | **Temperature(ºC)** | **Drying constants**  **(k,k_1_)** | **Drying coefficients (n,a,b,c,g,h,α,β,δ)** | **SSE** | **R^2^** | **RMSE** | **χ^2^** |
| --- | --- | --- | --- | --- | --- | --- | --- |
| Newton | 60 | 0.8379 |  | 0.0027 | 0.9972 | 0.0183 | 0.0004 |
|  | 65 | 1.0422 |  | 0.0021 | 0.9976 | 0.0172 | 0.0003 |
|  | 70 | 1.3372 |  | 0.0024 | 0.9970 | 0.0200 | 0.0005 |
| Average | | | | 0.0024 | 0.9973 | 0.0185 | 0.0004 |
| Page | 60 | 0.8349 | n=1.0174 | 0.0026 | 0.9973 | 0.0181 | 0.0004 |
|  | 65 | 1.0434 | n=1.0563 | 0.0018 | 0.9981 | 0.0160 | 0.0004 |
|  | 70 | 1.3854 | n=1.1497 | 0.0013 | 0.9987 | 0.0146 | 0.0003 |
| Average | | | | 0.0019 | 0.9980 | 0.0162 | 0.0004 |
| Modified Page | 60 | 0.8372 | n=1.0155 | 0.0026 | 0.9973 | 0.0181 | 0.0004 |
|  | 65 | 1.0411 | n=1.0563 | 0.0018 | 0.9981 | 0.0160 | 0.0004 |
|  | 70 | 1.3278 | n=1.1497 | 0.0013 | 0.9987 | 0.0146 | 0.0003 |
| Average | | | | 0.0019 | 0.9980 | 0.0162 | 0.0004 |
| Henderson and Pabis | 60 | 0.8448 | a=1.0071 | 0.0026 | 0.9973 | 0.0180 | 0.0004 |
|  | 65 | 1.0511 | a=1.0079 | 0.0020 | 0.9977 | 0.0170 | 0.0004 |
|  | 70 | 1.3481 | a=1.0083 | 0.0023 | 0.9970 | 0.0197 | 0.0006 |
| Average | | | | 0.0023 | 0.9973 | 0.0182 | 0.0005 |
| Midilli | 60 | 0.8496 | n=1.0409, a=1.0048, b=0.0022 | 0.0023 | 0.9975 | 0.0171 | 0.0006 |
|  | 65 | 1.0694 | n=1.0973, a=1.0028, b=0.0036 | 0.0013 | 0.9985 | 0.0137 | 0.0004 |
|  | 70 | 1.4327 | n=1.2022, a=1.0013, b=0.0048 | 0.0008 | 0.9990 | 0.0114 | 0.0004 |
| Average | | | | 0.0015 | 0.9983 | 0.0141 | 0.0005 |
| **Modified Midilli I** | **60** | **0.8681** | **n=1.0594, a=0.9890, b=0.0155** | **0.0022** | **0.9976** | **0.0167** | **0.0006** |
|  | **65** | **1.1043** | **n=1.1258, a=0.9830, b=0.0194** | **0.0012** | **0.9986** | **0.0130** | **0.0004** |
|  | **70** | **1.4940** | **n=1.2421, a=0.9811, b=0.0199** | **0.0006** | **0.9992** | **0.0101** | **0.0003** |
| **Average** | | | | **0.0013** | **0.9985** | **0.0133** | **0.0004** |
| Modified Midilli II | 60 | 0.8441 | n=1.0464, b=0.0023 | 0.0024 | 0.9975 | 0.0172 | 0.0005 |
|  | 65 | 1.0663 | n=1.1003, b=0.0036 | 0.0013 | 0.9984 | 0.0138 | 0.0003 |
|  | 70 | 1.4314 | n=1.2036, b=0.0048 | 0.0008 | 0.9990 | 0.0114 | 0.0003 |
| Average | | | | 0.0015 | 0.9983 | 0.0141 | 0.0004 |
| Logarithmic | 60 | 0.8651 | a=1.0012, c=0.0082 | 0.0025 | 0.9975 | 0.0166 | 0.0004 |
|  | 65 | 1.0715 | a=1.0022, c=0.0071 | 0.0020 | 0.9979 | 0.0157 | 0.0004 |
|  | 70 | 1.3608 | a=1.0050, c=0.0038 | 0.0024 | 0.9972 | 0.0186 | 0.0006 |
| Average | | | | 0.0023 | 0.9975 | 0.0170 | 0.0005 |
| Wang and Singh | 60 |  | a=−0.4904, b=0.0566 | 0.0593 | 0.9554 | 0.0861 | 0.0099 |
|  | 65 |  | a=−0.6013, b=0.0839 | 0.0566 | 0.9518 | 0.0899 | 0.0113 |
|  | 70 |  | a=−0.7680, b=0.1346 | 0.0505 | 0.9496 | 0.0917 | 0.0126 |
| Average | | | | 0.0555 | 0.9523 | 0.0893 | 0.0113 |
| Weibullian | 60 |  | α=1.1940, β=1.0174 | 0.0026 | 0.9973 | 0.0181 | 0.0004 |
|  | 65 |  | α=0.9606, β=1.0563 | 0.0018 | 0.9981 | 0.0160 | 0.0004 |
|  | 70 |  | α=0.7531, β=1.1497 | 0.0013 | 0.9987 | 0.0146 | 0.0003 |
| Average | | | | 0.0019 | 0.9980 | 0.0162 | 0.0004 |
| Weibullian I | 60 |  | n=1.0174, δ=2.7104 | 0.0026 | 0.9973 | 0.0181 | 0.0004 |
|  | 65 |  | n=1.0563, δ=2.1157 | 0.0018 | 0.9981 | 0.0160 | 0.0004 |
|  | 70 |  | n=1.1497, δ=1.5556 | 0.0013 | 0.9987 | 0.0146 | 0.0003 |
| Average | | | | 0.0019 | 0.9980 | 0.0162 | 0.0004 |
| Peleg | 60 |  | a=0.9160, b=0.8243 | 0.0086 | 0.9911 | 0.0328 | 0.0014 |
|  | 65 |  | a=0.7280, b=0.8304 | 0.0087 | 0.9900 | 0.0353 | 0.0017 |
|  | 70 |  | a=0.5578, b=0.8346 | 0.0091 | 0.9883 | 0.0389 | 0.0023 |
| Average | | | | 0.0088 | 0.9898 | 0.0357 | 0.0018 |
| Silva and others | 60 |  | a=0.9040, b=−0.0693 | 0.0025 | 0.9975 | 0.0176 | 0.0004 |
|  | 65 |  | a=1.2179, b=−0.1663 | 0.0015 | 0.9985 | 0.0148 | 0.0003 |
|  | 70 |  | a=1.8301, b=−0.4245 | 0.0009 | 0.9991 | 0.0126 | 0.0002 |
| Average | | | | 0.0016 | 0.9984 | 0.0150 | 0.0003 |
| Cubic | 60 |  | a=−0.0126, b=0.1616, c=−0.6796, d=0.9838 | 0.0062 | 0.9934 | 0.0278 | 0.0015 |
|  | 65 |  | a=−0.0231, b=0.2454, c=−0.8466, d=0.9883 | 0.0041 | 0.9951 | 0.0243 | 0.0014 |
|  | 70 |  | a=−0.0465, b=0.3967, c=−1.0892, d=0.9950 | 0.0027 | 0.9964 | 0.0214 | 0.0014 |
| Average | | | | 0.0044 | 0.9950 | 0.0245 | 0.0014 |
| Demir et al. | 60 | 0.9238 | n=0.9340, a=1.0019, b=0.0072 | 0.0025 | 0.9974 | 0.0176 | 0.0006 |
|  | 65 | 1.0306 | n=1.0346, a=1.0037, b=0.0053 | 0.0020 | 0.9977 | 0.0167 | 0.0007 |
|  | 70 | 1.1636 | n=1.1578, a=1.0085, b=−0.0002 | 0.0023 | 0.9970 | 0.0197 | 0.0012 |
| Average | | | | 0.0023 | 0.9974 | 0.0180 | 0.0008 |
| Binomil | 60 |  | a=0.0476, b=−0.4232, c=0.8991 | 0.0409 | 0.9564 | 0.0715 | 0.0082 |
|  | 65 |  | a=0.0712, b=−0.5214, c=0.9004 | 0.0403 | 0.9526 | 0.0759 | 0.0101 |
|  | 70 |  | a=0.1165, b=−0.6766, c=0.9091 | 0.0384 | 0.9502 | 0.0800 | 0.0128 |
| Average | | | | 0.0399 | 0.9531 | 0.0758 | 0.0104 |
| Two-term | 60 | k=0.8448, k_1_=0.8448 | a=4.3451, b=−3.3380 | 0.0026 | 0.9973 | 0.0180 | 0.0007 |
|  | 65 | k=1.0196, k_1_=1.0105 | a=4.3785, b=−3.3708 | 0.0021 | 0.9978 | 0.0163 | 0.0005 |
|  | 70 | k=1.1409, k_1_=1.0866 | a=4.3785, b=−3.3713 | 0.0024 | 0.9974 | 0.0184 | 0.0008 |
| Average | | | | 0.0024 | 0.9975 | 0.0176 | 0.0007 |
| Hii and others | 60 | k=0.8414, k_1_=0.8413 | n=1.0117, a=4.3408, b=−3.3351 | 0.0026 | 0.9974 | 0.0180 | 0.0009 |
|  | 65 | k=1.0469, k_1_=1.0467 | n=1.0505, a=4.3365, b=−3.3329 | 0.0019 | 0.9983 | 0.0155 | 0.0006 |
|  | 70 | k=1.3842, k_1_=1.3833 | n=1.1468, a=4.6997, b=−3.6978 | 0.0014 | 0.9988 | 0.0144 | 0.0007 |
| Average | | | | 0.0020 | 0.9981 | 0.0159 | 0.0007 |

**Table S5.** Drying constants and the drying coefficient of the selected thin layer drying models of steam blanched green mango slices.

| **Model** | **Temperature(ºC)** | **Drying constants**  **(k,k_1_)** | **Drying coefficients**  **(n,a,b,c,g,h,α,β,δ)** | **SSE** | **R^2^** | **RMSE** | **χ^2^** |
| --- | --- | --- | --- | --- | --- | --- | --- |
| Newton | 60 | 0.9253 |  | 0.0063 | 0.9944 | 0.0264 | 0.0008 |
|  | 65 | 1.0379 |  | 0.0039 | 0.9960 | 0.0222 | 0.0005 |
|  | 70 | 1.2510 |  | 0.0032 | 0.9965 | 0.0215 | 0.0005 |
| Average | | | | 0.0045 | 0.9957 | 0.0234 | 0.0006 |
| Page | 60 | 0.9057 | n=1.2217 | 0.0016 | 0.9989 | 0.0135 | 0.0002 |
|  | 65 | 1.0444 | n=1.0444 | 0.0023 | 0.9985 | 0.0168 | 0.0003 |
|  | 70 | 1.2767 | n=1.2767 | 0.0027 | 0.9979 | 0.0196 | 0.0004 |
| Average | | | | 0.0022 | 0.9985 | 0.0166 | 0.0003 |
| Modified Page | 60 | 0.9057 | n=1.2217 | 0.0018 | 0.9987 | 0.0141 | 0.0002 |
|  | 65 | 1.0386 | n=1.1465 | 0.0023 | 0.9985 | 0.0168 | 0.0003 |
|  | 70 | 1.2484 | n=1.1010 | 0.0027 | 0.9979 | 0.0196 | 0.0004 |
| Average | | | | 0.0022 | 0.9984 | 0.0168 | 0.0003 |
| Henderson and Pabis | 60 | 0.9450 | a=1.0203 | 0.0058 | 0.9945 | 0.0254 | 0.0008 |
|  | 65 | 1.0536 | a=1.0142 | 0.0037 | 0.9962 | 0.0215 | 0.0006 |
|  | 70 | 1.2607 | a=1.0076 | 0.0032 | 0.9966 | 0.0213 | 0.0006 |
| Average | | | | 0.0042 | 0.9958 | 0.0227 | 0.0007 |
| Midilli | 60 | 0.9138 | n=1.2549, a=0.9969, b=0.0027 | 0.0008 | 0.9993 | 0.0092 | 0.0002 |
|  | 65 | 1.0759 | n=1.1982, a=1.0025, b=0.0044 | 0.0008 | 0.9992 | 0.0100 | 0.0002 |
|  | 70 | 1.3376 | n=1.1811, a=1.0018, b=0.0062 | 0.0010 | 0.9988 | 0.0118 | 0.0003 |
| Average | | | | 0.0008 | 0.9991 | 0.0103 | 0.0002 |
| **Modified Midilli I** | **60** | **0.9313** | **n=1.2719, a=0.9824, b=0.0142** | **0.0008** | **0.9992** | **0.0097** | **0.0002** |
|  | **65** | **1.1215** | **n=1.2411, a=0.9782, b=0.0238** | **0.0006** | **0.9994** | **0.0084** | **0.0001** |
|  | **70** | **1.4257** | **n=1.2467, a=0.9718, b=0.0293** | **0.0006** | **0.9993** | **0.0092** | **0.0002** |
| **Average** | | | | **0.0007** | **0.9993** | **0.0091** | **0.0002** |
| Modified Midilli II | 60 | 0.9176 | n=1.2501, b=0.0027 | 0.0008 | 0.9993 | 0.0092 | 0.0001 |
|  | 65 | 1.0730 | n=1.2014, b=0.0044 | 0.0008 | 0.9991 | 0.0101 | 0.0002 |
|  | 70 | 1.3356 | n=1.1831, b=0.0062 | 0.0010 | 0.9988 | 0.0118 | 0.0002 |
| Average | | | | 0.0009 | 0.9991 | 0.0104 | 0.0002 |
| Logarithmic | 60 | 0.9402 | a=1.0218, c=−0.0020 | 0.0058 | 0.9945 | 0.0254 | 0.0010 |
|  | 65 | 1.0748 | a=1.0082, c=0.0075 | 0.0035 | 0.9962 | 0.0210 | 0.0007 |
|  | 70 | 1.3082 | a=0.9958, c=0.0141 | 0.0026 | 0.9969 | 0.0193 | 0.0007 |
| Average | | | | 0.0040 | 0.9959 | 0.0219 | 0.0008 |
| Wang and Singh | 60 |  | a=−0.4700, b=0.0496 | 0.1069 | 0.9302 | 0.1090 | 0.0153 |
|  | 65 |  | a=−0.5369, b=0.0653 | 0.0955 | 0.9296 | 0.1092 | 0.0159 |
|  | 70 |  | a=−0.6431, b=0.0934 | 0.0928 | 0.9216 | 0.1152 | 0.0186 |
| Average | | | | 0.0984 | 0.9271 | 0.1111 | 0.0166 |
| Weibullian | 60 |  | α=1.0845, β=1.2217 | 0.0016 | 0.9989 | 0.0135 | 0.0002 |
|  | 65 |  | α=0.9628, β=1.1465 | 0.0023 | 0.9985 | 0.0168 | 0.0004 |
|  | 70 |  | α=0.8010, β=1.1010 | 0.0027 | 0.9979 | 0.0196 | 0.0005 |
| Average | | | | 0.0022 | 0.9985 | 0.0166 | 0.0004 |
| Weibullian I | 60 |  | n=1.2217, δ=2.1464 | 0.0016 | 0.9989 | 0.0135 | 0.0002 |
|  | 65 |  | n=1.1465, δ=1.9929 | 0.0023 | 0.9985 | 0.0168 | 0.0004 |
|  | 70 |  | n=1.1010, δ=1.7086 | 0.0027 | 0.9979 | 0.0196 | 0.0005 |
| Average | | | | 0.0022 | 0.9985 | 0.0166 | 0.0004 |
| Peleg | 60 |  | a=0.8004, b=0.8459 | 0.0257 | 0.9760 | 0.0535 | 0.0037 |
|  | 65 |  | a=0.7024, b=0.8538 | 0.0172 | 0.9820 | 0.0463 | 0.0029 |
|  | 70 |  | a=0.5620, b=0.8678 | 0.0123 | 0.9854 | 0.0418 | 0.0025 |
| Average | | | | 0.0184 | 0.9812 | 0.0472 | 0.0030 |
| Silva and others | 60 |  | a=1.2909, b=−0.3662 | 0.0021 | 0.9983 | 0.0153 | 0.0003 |
|  | 65 |  | a=1.4072, b=−0.3462 | 0.0017 | 0.9991 | 0.0146 | 0.0003 |
|  | 70 |  | a=1.6099, b=−0.3141 | 0.0023 | 0.9985 | 0.0181 | 0.0005 |
| Average | | | | 0.0020 | 0.9986 | 0.0160 | 0.0003 |
| Cubic | 60 |  | a=−0.0107, b=0.1535, c=−0.6907, d=0.9833 | 0.0043 | 0.9959 | 0.0219 | 0.0009 |
|  | 65 |  | a=−0.0164, b=0.2022, c=−0.7848, d=0.9812 | 0.0064 | 0.9932 | 0.0283 | 0.0016 |
|  | 70 |  | a=−0.0292, b=0.2968, c=−0.9489, d=0.9801 | 0.0069 | 0.9917 | 0.0314 | 0.0023 |
| Average | | | | 0.0059 | 0.9936 | 0.0272 | 0.0016 |
| Demir et al. | 60 | 0.9643 | n=0.9750, a=1.0218, b=−0.0020 | 0.0058 | 0.9945 | 0.0254 | 0.0012 |
|  | 65 | 1.0347 | n=1.0387, a=1.0082, b=0.0075 | 0.0035 | 0.9962 | 0.0210 | 0.0009 |
|  | 70 | 1.1466 | n=1.1409, a=0.9958, b=0.0141 | 0.0026 | 0.9969 | 0.0193 | 0.0009 |
| Average | | | | 0.0040 | 0.9959 | 0.0219 | 0.0010 |
| Binomil | 60 |  | a=0.0409, b=−0.3948, c=0.8677 | 0.0726 | 0.9309 | 0.0898 | 0.0121 |
|  | 65 |  | a=0.0538, b=−0.4509, c=0.8709 | 0.0654 | 0.9305 | 0.0904 | 0.0131 |
|  | 70 |  | a=0.0767, b=−0.5380, c=0.8690 | 0.0647 | 0.9225 | 0.0961 | 0.0162 |
| Average | | | | 0.0675 | 0.9279 | 0.0921 | 0.0138 |
| Two-term | 60 | k=1.4926 k1=1.7718 | a=4.2877, b=−3.2891 | 0.0016 | 0.9989 | 0.0133 | 0.0003 |
|  | 65 | k=1.5901 k1=1.8471 | a=4.1927, b=−3.1901 | 0.0024 | 0.9986 | 0.0172 | 0.0006 |
|  | 70 | k=1.2202 k1=1.2083 | a=4.3719, b=−3.3644 | 0.0032 | 0.9966 | 0.0213 | 0.0011 |
| Average | | | | 0.0024 | 0.9981 | 0.0172 | 0.0007 |
| Hii and others | 60 | k=1.3214 k₁=1.5163 | n=1.0932, a=3.9654, b=−2.9672 | 0.0015 | 0.9991 | 0.0131 | 0.0004 |
|  | 65 | k=5.5568 k₁=4.5797 | n=0.5333, a=4.6653, b=−3.6653 | 0.0004 | 0.9996 | 0.0073 | 0.0001 |
|  | 70 | k=1.2802 k₁=1.2803 | n=1.0981, a=4.3326, b=−3.3297 | 0.0027 | 0.9980 | 0.0195 | 0.0013 |
| Average | | | | 0.0015 | 0.9989 | 0.0133 | 0.0006 |

**Table S6.** ANOVA results for the effect of pretreatment and drying temperature on techno-functional properties, total phenolic content, and organoleptic characteristics

| **Bulk Density** | | | | |
| --- | --- | --- | --- | --- |
| **Source of variation** | **df** | **F** | **p-value** | **F crit** |
| **Temperature** | 2 | 3.242909 | **0.056671** | 3.402826 |
| **Pretreatment** | 3 | 4.643636 | **0.010685** | 3.008787 |
| **Interaction** | 6 | 0.819636 | **0.565646** | 2.508189 |
| **Water absorption capacity** | | | | |
| **Temperature** | 2 | 198.0359 | **1.21E-15** | 3.402826 |
| **Pretreatment** | 3 | 121.4494 | **1.21E-14** | 3.008787 |
| **Interaction** | 6 | 32.23534 | **2.4E-10** | 2.508189 |
| **Oil absorption capacity** | | | | |
| **Temperature** | 2 | 26.18167 | **9.29E-07** | 3.402826 |
| **Pretreatment** | 3 | 79.9586 | **1.24E-12** | 3.008787 |
| **Interaction** | 6 | 2.616956 | **0.042736** | 2.508189 |
| **Total Phenolic content** | | | | |
| **Temperature** | 2 | 55.1128 | **1.07E-09** | 3.402826 |
| **Pretreatments** | 3 | 69.53216 | **5.58E-12** | 3.008787 |
| **Interaction** | 6 | 0.648186 | **0.691117** | 2.508189 |
| **Organoleptic characteristic (Among Pretreatments)** | | | | |
| **Color** | 3 | 7.578259 | **0.000113** | 2.682809 |
| **Flavor** | 3 | 4.59955 | **0.004439** | 2.682809 |

**p <* 0.05 denotes statistically significant difference

**Fig. S1.** Comparison and validation of experimental moisture ratio and predicted moisture ratio using the best fitted drying model for control at different drying temperature: (A) 60 ºC, (B) 65 ºC, and (C) 70 ºC

A

B

C

**Fig. S2.** Comparison and validation of experimental moisture ratio and predicted moisture ratio using the best fitted drying model for 1% citric acid treated samples at different drying temperature: (A) 60 ºC, (B) 65 ºC, and (C) 70 ºC

A

B

C

**Fig. S3.** Comparison and validation of experimental moisture ratio and predicted moisture ratio using the best fitted drying model for hot water blanched samples at different drying temperature: (A) 60 ºC, (B) 65 ºC, and (C) 70 ºC

A

B

C

**Fig. S4.** Comparison and validation of experimental moisture ratio and predicted moisture ratio using the best fitted drying model for steam blanched samples at different drying temperature: (A) 60 ºC, (B) 65 ºC, and (C) 70 ºC

A

B

C

**Reference:**

Basset-Mens, C., Payen, S., Vannière, H., Braun, A., & Biard, Y. (2018). Life cycle assessment of mango systems. In Victor Galan Sauco & Ping Lu (Eds.), *Achieving sustainable cultivation of mangoes* (pp.429–456). Burleigh Dodds Science Publishing. https://doi.org/10.19103/as.2017.0026.18

Ram, R., & Verma, A.K. (2015). Energy input, output and economic analysis inorganic production of mango (*Mangifera indica*) cv. Dashehari. *The Indian Journal of Agricultural Sciences*. <https://api.semanticscholar.org/CorpusID:83002709>

World Population Review (2025). *Mango Production by Country 2025*. <https://www.Fao.Org/Faostat/En/#data/QCL>. https://worldpopulationreview.com/country-rankings/mango-production-by-country?utm_source=chatgpt.com
